# 2,4‐Dihydroxybenzoic Acid, a Novel SA Derivative, Controls Plant Immunity via UGT95B17‐Mediated Glucosylation: A Case Study in *Camellia Sinensis*

**DOI:** 10.1002/advs.202307051

**Published:** 2023-12-08

**Authors:** Mengqian Lu, Yifan Zhao, Yingying Feng, Xiaoyan Tang, Wei Zhao, Keke Yu, Yuting Pan, Qiang Wang, Jilai Cui, Mengting Zhang, Jieyang Jin, Jingming Wang, Mingyue Zhao, Wilfried Schwab, Chuankui Song

**Affiliations:** ^1^ State Key Laboratory of Tea Plant Biology and Utilization International Joint Laboratory on Tea Chemistry and Health Effects Anhui Agricultural University Hefei Anhui 230036 P. R. China; ^2^ Key Laboratory of Tea Plant Biology of Henan Province College of Life Science Xinyang Normal University 237 Nanhu R. Xinyang Henan 464000 P. R. China; ^3^ Biotechnology of Natural Products Technische Universität München Liesel‐Beckmann‐Str. 1 85354 Freising Germany

**Keywords:** 2,4‐DHBA, Camellia sinensis, pathogen resistance, UDP‐glucosyltransferase

## Abstract

The plant hormone salicylic acid (SA) plays critical roles in plant innate immunity. Several SA derivatives and associated modification are identified, whereas the range and modes of action of SA‐related metabolites remain elusive. Here, the study discovered 2,4‐dihydroxybenzoic acid (2,4‐DHBA) and its glycosylated form as native SA derivatives in plants whose accumulation is largely induced by SA application and *Ps*. *camelliae‐sinensis* (*Pcs*) infection. CsSH1, a 4/5‐hydroxylase, catalyzes the hydroxylation of SA to 2,4‐DHBA, and UDP‐glucosyltransferase UGT95B17 catalyzes the formation of 2,4‐DHBA glucoside. Down‐regulation reduced the accumulation of 2,4‐DHBA glucosides and enhanced the sensitivity of tea plants to *Pcs*. Conversely, overexpression of *UGT95B17* increased plant disease resistance. The exogenous application of 2,4‐DHBA and 2,5‐DHBA, as well as the accumulation of DHBA and plant resistance comparison, indicate that 2,4‐DHBA functions as a potentially bioactive molecule and is stored mainly as a glucose conjugate in tea plants, differs from the mechanism described in *Arabidopsis*. When 2,4‐DHBA is applied exogenously, *UGT95B17*‐silenced tea plants accumulated more 2,4‐DHBA than SA and showed induced resistance to *Pcs* infection. These results indicate that 2,4‐DHBA glucosylation positively regulates disease resistance and highlight the role of 2,4‐DHBA as potentially bioactive molecule in the establishment of basal resistance in tea plants.

## Graphical abstract

The plant hormone SA plays critical roles in plant innate immunity, whereas the range and mechanisms of action of SA‐related metabolites remain elusive. The current study identified 2,4‐dihydroxybenzoic acid as a naturally occurring derivative of SA in plants. This compound exhibits potential bioactivity in regulating disease resistance through UGT95B17 mediated glucosylation in tea plants.

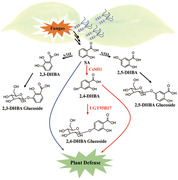

## 1 Introduction

Plants are severely affected by pathogenic viruses, bacteria, fungi, and oomycetes, which cause destructive diseases during growing in fields. In order to effectively prevent infection, plants have developed a multilayered immune system that protects local tissues from infection by the majority of pathogens and primes systemic tissues against subsequent attack.^[^ ^1^ ^]^ This plant immune system is extremely complex and possesses a two‐tiered innate immune system called pattern‐triggered immunity and effector‐triggered immunity.^[^ ^2^ ^]^ The structural range of secondary metabolites produced by plants during defense activation is vast, among them, SA plays a vital role in leaf senescence, flowering, and thermogenesis, and SA is essential for basal resistance to virulent pathogens and for the establishment of systemic acquired resistance.^[^ ^3^ ^]^

SA homeostasis directly affects its biological functions. Although a small amount of SA produced in planta remains in a free state, most SA undergoes a series of complex biological modifications including glucosylation, methylation, amino acid (AA) conjugation, and hydroxylation. In plants, SA is rapidly metabolized to SA 2‐O‐β‐D‐glucoside (SAG) and SA glucose ester (SGE) for transport and storage.^[^ ^4^ ^]^ A number of SA derivatives and genes regulating SA modification steps also appear to be important for plant resistance. MeSA, the methylated derivative of SA, has been associated with several aspects of plant defense signaling.^[^ ^5^ ^]^ AA conjugation of SA at trace levels was found in infected *Arabidopsis* plants.^[^ ^6^ ^]^ Recently, high levels of 2,3‐ and 2,5‐dihydroxybenzoic acid (2,3‐DHBA and 2,5‐DHBA, respectively) have been detected as sugar conjugates produced at even higher levels than SA and its glycosides, suggesting that hydroxylation is an important modification for SA catabolism.^[^ ^7^ ^]^

Recently, the enzymes catalyzing SA to 2,3‐DHBA (S3H) and to 2,5‐DHBA (S5H) have been identified, and SA content accumulated in *s3h* or *s5h* mutant.^[^ ^7^ ^]^ A large amount of 2,3‐DHBA and 2,5‐DHBA in the form of glycoside conjugates was detected in infected and aged leaves in *Arabidopsis*.^[^ ^8^ ^]^ 2,5‐DHBA is one of the most widely produced aromatic acids in green plants, and its formation is largely induced by the citrus exocortis viroid and tomato mosaic virus in tomato, at much higher levels than SA.^[^ ^9^ ^]^ Besides, 2,5‐DHBA is identified as a pathogen signal for plant defense activation that induces pathogenesis‐related (PR) proteins.^[^ ^9a^ ^]^ Because of the cytotoxicity of DHBA, 2,3‐DHBA and 2,5‐DHBA are always present as sugar conjugates in plants, and the enzymes involved in the formation of 2,3‐DHBA and 2,5‐DHBA glycosides were recently identified in *Arabidopsis*.^[^ ^10^ ^]^ Although some glycosyltransferases capable of catalyzing the transformation of 2,4‐DHBA in vitro to form their glucose conjugates have been identified,^[^ ^11^ ^]^ it remains unclear whether 2,4‐DHBA glucose conjugates occur naturally in plants, and if so, their formation and physiological functions are still unknown.

Tea is valued for its leaf buds and young leaves, from which the tea beverage is produced and is widely planted all over the world. The diseases of tea plants are mainly caused by fungi, which result in severe economic losses. Gray blight is instigated by *Pestalotiopsis*‐like species,^[^ ^12^ ^]^ and *Pseudopestalotiopsis camelliae‐sinensis* (*Pcs*) has been isolated from tea gray blight.^[^ ^4b^ ^]^ Here, we reported the discovery of 2,4‐DHBA and its glycosylated form as native SA derivatives in plants. UDP‐glucosyltransferase UGT95B17 catalyzes the formation of 2,4‐DHBA glucoside in addition to 2,3‐DHBA and 2,5‐DHBA in vitro. Down‐regulation reduced the accumulation of 2,4‐DHBA and 2,5‐DHBA glucosides and enhanced the sensitivity of tea plants to *Pcs*. After exogenous application of 2,4‐DHBA and 2,5‐DHBA, DHBA analyses, and comparison of plant resistance, we demonstrated that 2,4‐DHBA functions as a potentially bioactive molecule and is stored mainly as a glucose conjugate in tea plants. When 2,4‐DHBA was applied, *UGT95B17*‐silenced tea plants accumulated more 2,4‐DHBA, and *UGT95B17*‐silenced tea plants showed induced resistance to *Pcs* infection. Interestingly, when 2,4‐DHBA was exogenously applied to *UGT95B17*‐silenced tea plants, less SA was detected, and tea plants exhibited enhanced resistance to *Pcs*. All in all, our results suggest that UGT95B17 mediated 2,4‐DHBA glucosylation positively regulates disease resistance and highlights the role of 2,4‐DHBA as potentially bioactive molecule in the establishment of basal resistance.

## 2 Results

### 2.1 Fungal Infection Triggers the Formation of Several DHBA Sugar Conjugates

**Figure 1:**
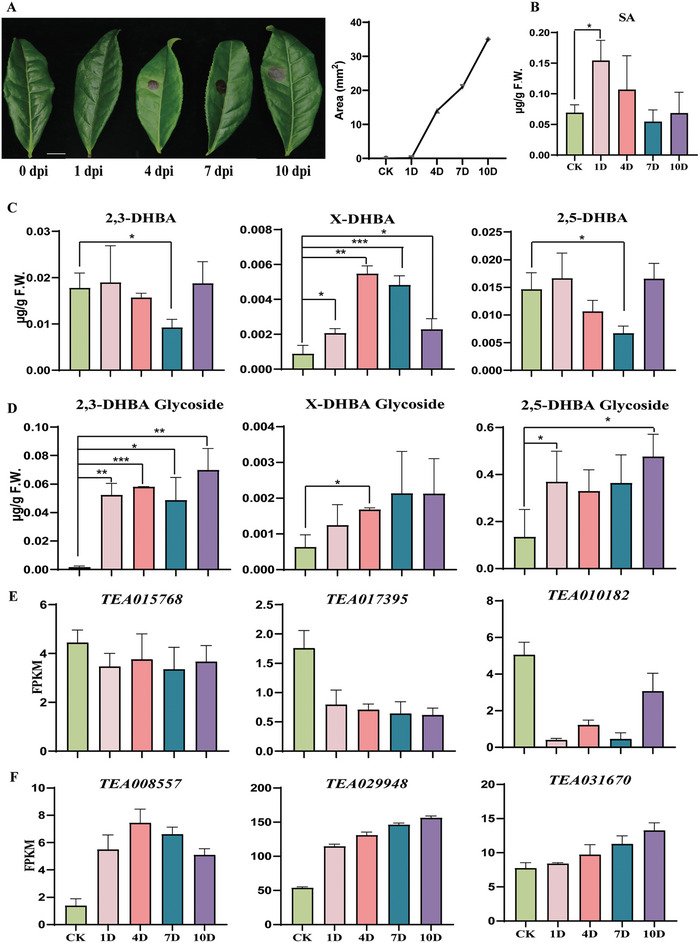
Fungal infection triggers the formation of several DHBA sugar conjugates in tea plants. A) The phenotype of tea leaves and lesion statistics chart after fungal infection. Bar = 1 cm. B) The relative content of SA after *Pcs* infection. C,D) The relative contents of DHBA and DHBA glycosides after *Pcs* infection. Control, inoculated with pure water. An unknown compound was identified as a novel DHBA sugar conjugate (x‐DHBA Glc) due to its similarity in mass spectrum fragment with the conjugated forms of 2,3‐ and 2,5‐DHBA. Data are presented as mean ± sd of at least three replicates. E) Homologous genes expression of UGT89A2 (*TEA015768*, *TEA017395*, *TEA010182*). F, Homologous genes expression of UGT76D1 (*TEA008557*, *TEA029948*, *TEA031670*).

As hydroxylation is an important modification for SA catabolism,^[^ ^7^ ^]^ the changes of 2,3‐DHBA, 2,5‐DHBA and their corresponding sugar conjugates in tea leaves after infection with the tea pathogen *Pcs* were examined for 0, 1, 4, 7 and 10 dpi (Figure **1**A) by liquid chromatography–mass spectrometry (LC–MS). The level of SA increased significantly to 2.22 times that of the control at 1 dpi after inoculation and then decreased to normal levels at 7, 10 dpi (Figure 1B). The content of 2,3‐, 2,5‐DHBA increased by 1.07, 1.13‐fold at 1 dpi post‐inoculation compared to the control, followed by a notable decline at 7 dpi and subsequent restoration to normal leaf levels at 10 dpi (Figure 1C). In contrast, the levels of 2,3‐ and 2,5‐DHBA sugar conjugates increased rapidly at 1 dpi and remained consistent over the following 10 days (Figure 1D).

An unknown compound was tentatively identified as a novel DHBA sugar conjugate (x‐DHBA) because it shares a similar mass spectrum fragment with the conjugated form of 2,3‐ and 2,5‐DHBA. In contrast to the changes observed in 2,3‐DHBA and 2,5‐DHBA sugar conjugate, x‐DHBA and its putative glucoside were significantly induced by *Pcs* infection, and their accumulation were sustained induction for 7 days (Figure 1C,D). These results suggested that 2,3‐DHBA, 2,5‐DHBA and especially the unknown x‐DHBA sugar conjugate might be involved in plant defense responses. This prompted us to find the enzymes/ genes involved in the formation of these DHBA sugar conjugates, to structurally characterize the unknown DHBA‐conjugated form, and to ultimately reveal their roles in plant defense.

### 2.2 Homologous UGT89A2 and UGT76D1 had no DHBA Glycosylation Activity

As UGT76D1 could convert 2,3‐DHBA and 2,5‐DHBA to 2,3‐DHBA and 2,5‐DHBA glucosides,^[^ ^10a^ ^]^ and UGT89A2 produced 2,3‐DHBA or 2,5‐DHBA xylosides in *Arabidopsis*,^[^ ^10^, ^13^ ^]^ the expression levels and function of the homologous genes of UGT89A2 and UGT76D1 in tea plants were analyzed. Three homologous UGTs genes of UGT89A2 were found in tea plants (*TEA015768*, *TEA017395*, and *TEA010182*) and their transcription levels were not induced by pathogens (Figure 1E). Three homologous genes of UGT76D1 (*TEA008557*, *TEA029948*, and *TEA031670*) were induced and showed a similar expression response to *Pcs* infection (Figure 1F). Therefore, these 3 UGTs were successfully cloned from tea plants and expressed in *Escherichia coli* BL21(DE3) pLysS strain. However, no products were formed when 2,3‐, 2,4‐, 2,5‐, 2,6‐DHBA were used as substrates with different sugar donors (Table S5, Supporting Information). The results showed that the homologous UGT89A2 and UGT76D1 proteins lacked DHBA glycosylation activity, suggesting that yet unknown UGTs catalyze the formation of DHBA sugar conjugates in tea plants.

### 2.3 Candidate Genes were Identified by Correlating UGTs Expression and DHBA Sugar Conjugate Content During Pathogen Infection

**Figure 2:**
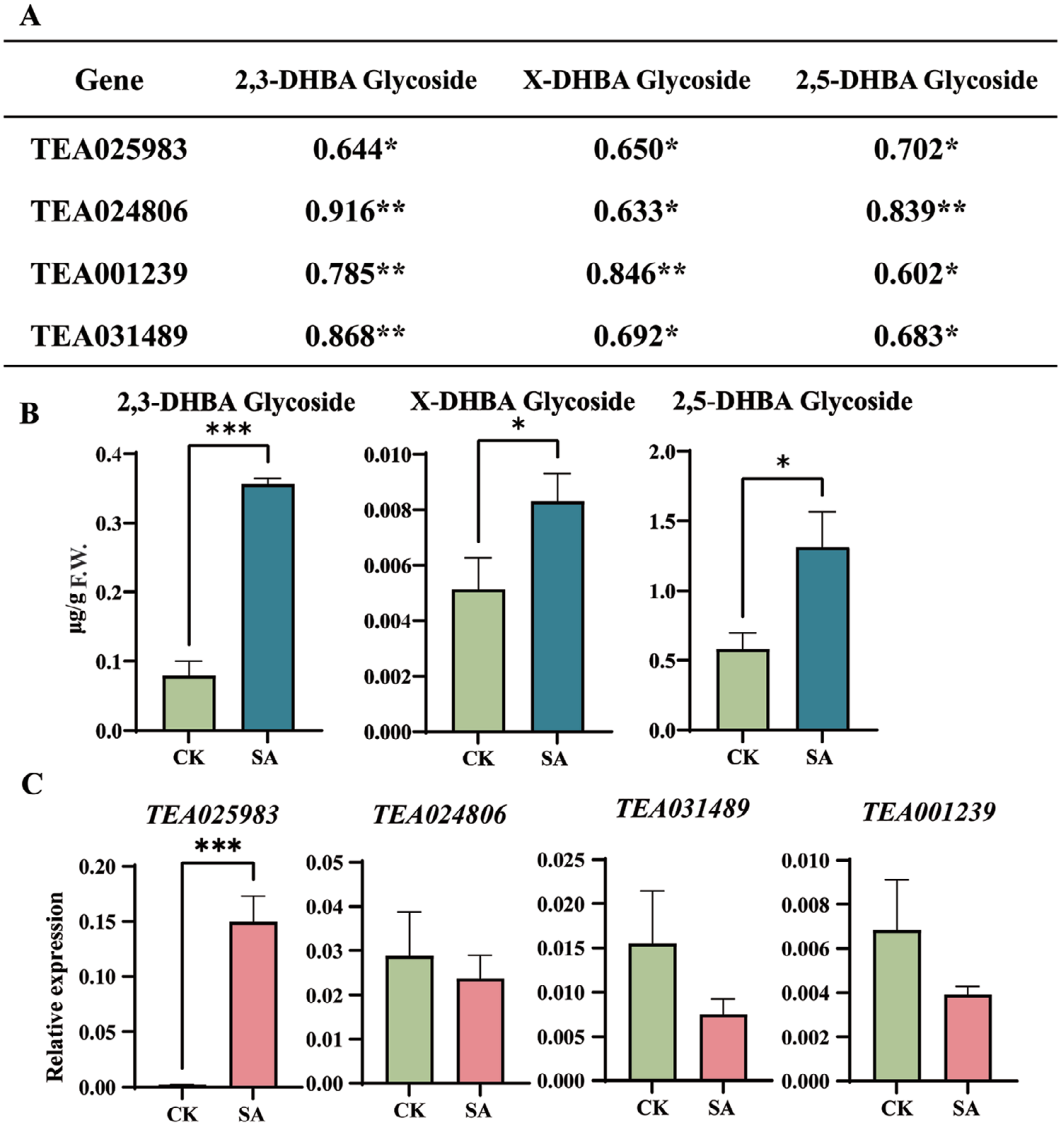
Candidate UGTs identified by gene expression and DHBA sugar conjugate correlation analysis during pathogen infection. A) A correlation analysis between the expression levels of putative UGT genes and the concentration of 2,3‐, X‐, 2,5‐DHBA glycosides response to *Pcs* infection. Numbers represent the correlation coefficient. ^*^ *p* < 0.05, ^**^ *p* < 0.01. B) The relative contents of DHBA glucosides after SA treatment. C) The expression of putative genes by SA treatment.

To find the candidate UGTs involved in the formation of DHBA sugar conjugate during pathogen infection, tea leaves infected by *Pcs* for 0, 1, 4, 7, 10 dpi were collected for RNA sequencing. Eighty putative UGTs were found whose transcript levels were significantly induced by pathogens in tea leaves (Table S1, Supporting Information). To mine UGT genes associated with the formation of DHBA sugar conjugates, correlation analysis was performed between the transcripts of these 80 UGTs and the content of 2,3‐DHBA, 2,5‐DHBA, and the unknown x‐DHBA glycosides of the tea plant during pathogen infection (Figure 1D; Table S2, Supporting Information). Four UGTs (*TEA025983*, *TEA024806*, *TEA001239*, *TEA031489*) showed significant positive correlation with the content of 2,3‐, 2,5‐, and the unknown x‐DHBA glycoside (**Figure** **2**A), therefore, these four UGTs were selected as candidates for further study.

Because 2,3 and 2,5‐DHBA are hydroxylated products of SA in planta, we infer that the content these DHBA glycosides and the involved genes should be induced after exogenous feeding of SA. As expected, the content of 2,3‐, 2,5‐, and the unknown x‐DHBA glycoside was significantly induced in tea plants when SA was applied (Figure 2B), indicating that the unknown x‐DHBA was also a SA derivative in tea plants. Gene expression of the four selected UGTs was detected by qRT‐PCR. The transcript level of *TEA025983* was very low in the control tea leaves but its expression was strongly induced by SA, whereas the transcripts of *TEA024806*, *TEA001239* and *TEA031489* were almost not altered after application of SA compared to the control (Figure 2C). These results indicated that TEA025983 might be involved in the biosynthesis of DHBA glycoside(s) in tea plants.

### 2.4 UGT95B17 Catalyzes 2,3‐, 2,4‐, and 2,5‐DHBA Glycosylation In Vitro

**Figure 3:**
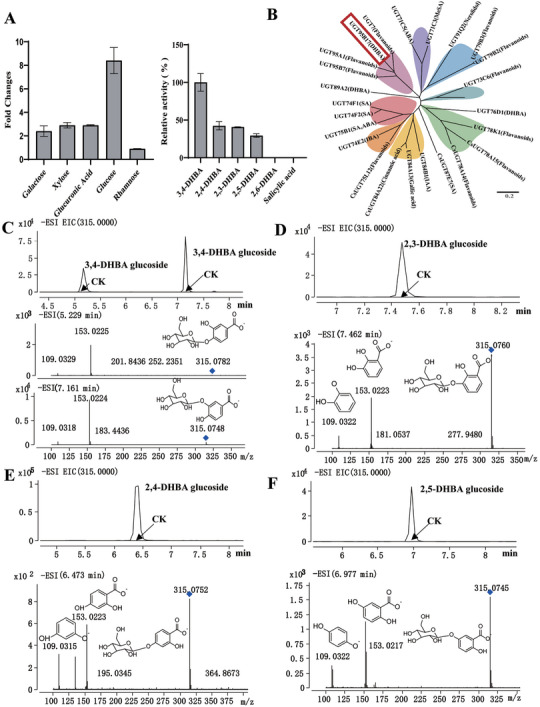
Enzymatic analysis with different substrates and identification of enzymatically formed products by LC–MS. A) Sugar specific screening UGT95B17 with different sugar donors. Activity screening of UGT95B17 protein with different substrates. The activity of 3,4‐DHBA was set as 100%. Values are expressed as the mean ± standard deviation of triplicate samples. B) Phylogenetic analysis of UGT95B17 and other identified functional UGTs. Product formed from C) 3,4‐DHBA, D) 2,3‐DHBA, E) 2,4‐DHBA, and F) 2,5‐DHBA when the substrates were incubated with UDP‐glucose and recombinant UGT95B17 and empty vector control (CK).

To further verify whether TEA025983 is involved in the biosynthesis of DHBA glycoside(s), the corresponding gene of *TEA025983* was cloned from tea plant, expressed in *Escherichia coli* BL21(DE3) and purified successfully. The protein was assigned to UGT95B17 by the UGT Nomenclature Committee.^[^ ^14^ ^]^ The enzymatic activity of purified UGT95B17 was tested by UDP‐Glo glycosyltransferase assay. SA, 2,3‐, 2,5‐, and further commercially available DHBAs were used as substrates (**Figure** **3**A). When UDP‐glucose was used as donor substrate, UGT95B17 readily glycosylated a range of DHBAs, including 3,4‐DHBA, 2,3‐DHBA, 2,4‐DHBA, 2,5‐DHBA and THPA (2′,4′,6′‐Trihydroxyacetophenone) (Figure 3A; Figure S1, Supporting Information). The sugar donor specificity of UGT95B17 was performed with different sugar donors. UGT95B17 preferred UDP‐glucose as sugar donor over UDP‐xylose, UDP‐galactose, UDP‐glucuronic acid, and UDP‐rhamnose (Figure 3A).

### 2.5 Kinetic Properties of the Recombinant UGT95B17 Enzyme

The highest activity of UGT95B17 was detected at pH 7.5 (Figure S2A, Supporting Information) and 25 °C (Figure S2C, Supporting Information). Kinetic properties were determined in the linear range of the enzymatic reaction (40 min reaction time) at optimized conditions (Figure S2B, Supporting Information). The apparent K_M_ value for 2,3‐DHBA, 2,4‐DHBA and 2,5‐DHBA was 3.31 ± 0.58, 0.81±0.26, and 0.31 ± 0.11 µm, respectively (Figure S2D–F, Supporting Information). The low Km value for DHBA strongly suggests that 2,3‐DHBA, 2,4‐DHBA, and 2,5‐DHBA are the in vivo substrate of UGT95B17, indicating that UGT95B17 is one of the enzymes involved in the formation of DHBA glucosides.

### 2.6 2,4‐DHBA and Its Glucoside were Unambiguously Discovered as Novel SA Derivative in Tea Plants

**Figure 4:**
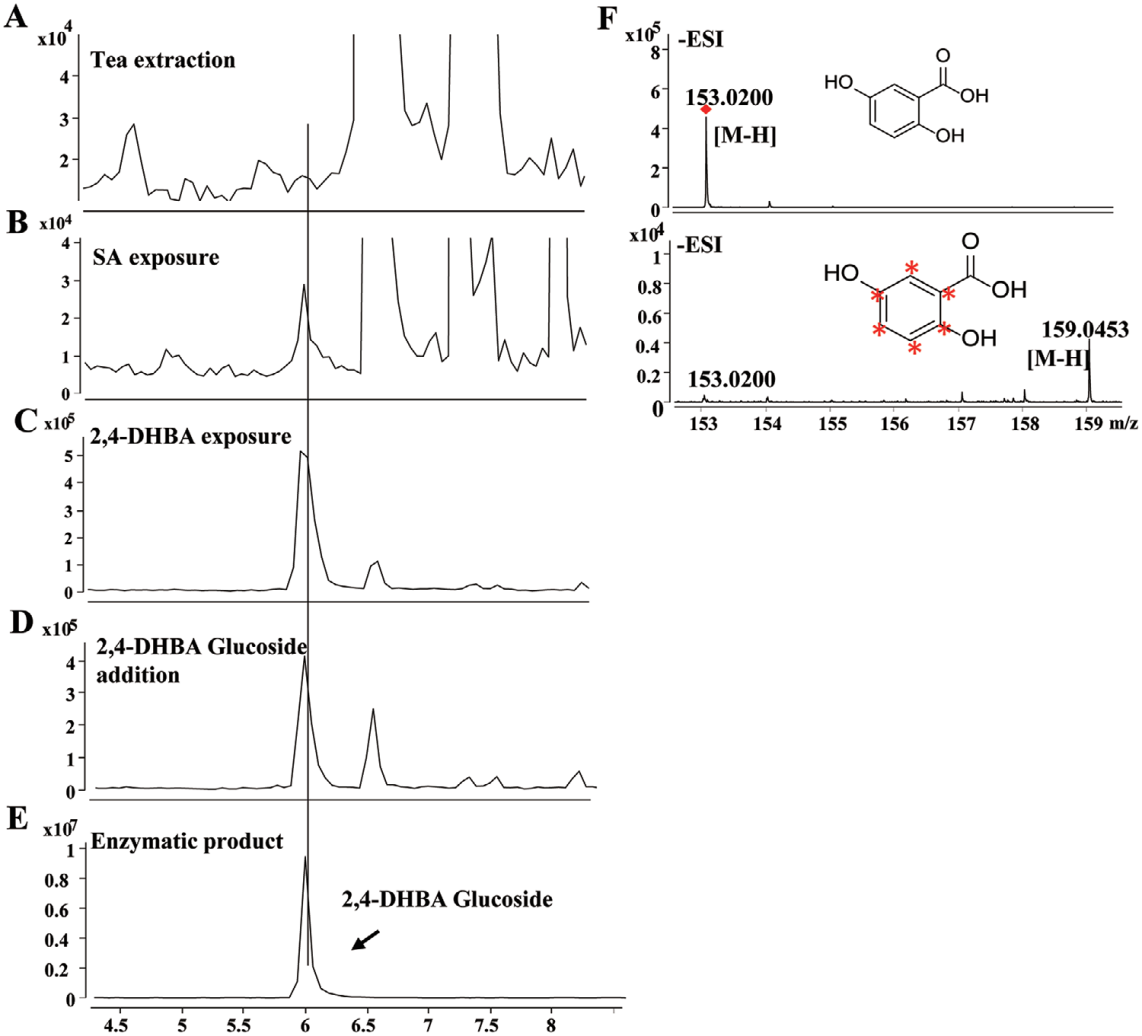
2,4‐DHBA glucoside was identified and labeled 2,4‐DHBA was detected after feeding [^13^C_6_] SA in tea plants. The identity of 2,4‐DHBA glucoside in untreated leaves (A), tea leaves exposed to 5 mm SA (B) and 2,4‐DHBA (C) for 24 h, and 2,4‐DHBA glucoside addition (D) was confirmed by LC–MS. F, The identity of labeled 2,4‐DHBA from tea leaves fed with water as control and [^13^C_6_] SA.

To characterize the structure of the DHBA sugar conjugates in tea leaves, the retention time and accurate mass of the detected DHBA sugar conjugates were compared with those of the enzymatic products and commercially available references. As expected, 2,3‐DHBA, 2,5‐DHBA sugar conjugates were confirmed as 2,3‐DHBA‐glucoside and 2,5‐DHBA‐glucoside, respectively. The x‐DHBA derivative with a retention time of 6.0 min was identified as 2,4‐DHBA glucoside by comparing the retention time and accurate mass with those of the enzyme product of UGT95B17 (**Figure** **4**A,E). 2,4‐DHBA glucoside was further confirmed as a novel SA derivative, as its content was significantly induced by exogenous feeding of SA (Figure 4B). To prove that 2,4‐DHBA can be glycosylated in tea plants, undamaged tea leaves were exposed to 2,4‐DHBA and LC‐MS analysis confirmed that the concentration of 2,4‐DHBA glucoside significantly increased after exposure for 24 h (Figure 4C). Furthermore, when enzymatically synthesized 2,4‐DHBA glucoside was added to the tea sample, the concentration of 2,4‐DHBA glucoside increased compared with that in the control. This further confirmed the structure of the unknown x‐DHBA sugar conjugates (Figure 4D). 2,4‐DHBA and its glucoside have not been reported in plants until now. To further investigate the potential derivative of 2,4‐DHBA from SA, we conducted an isotope tracer experiment with SA‐^13^C_6_ (600 µL, 300 µm). The results showed that isotope‐labeled 2,4‐DHBA can be detected after 24 h in tea leaves fed with SA‐^13^C_6_, demonstrating that tea plants can convert SA into 2,4‐DHBA (Figure 4F).

### 2.7 *CsSH1* and *CsSH2* Can be Induced by *Pcs* and SA

Isotope tracing methods have revealed the conversion of SA to 2,4‐DHBA (Figure 4F); however, the enzyme (s) involved in the formation of 2,4‐ DHBA remain unidentified. In *Arabidopsis*, S3H and S5H enzymes are known to convert SA into 2,3‐DHBA and 2,5‐DHBA, respectively.^[^ ^7^ ^]^ In tea plants, the homologous genes CsSH1 (TEA002405) and CsSH2 (TEA027220) have been identified. Sequence alignment analysis has confirmed that both CsSH1 and CsSH2 possess a conserved catalytic domain called Pfam PF03171 and belong to the 2‐oxoglutarate–Fe(II) oxygenase family of enzymes (Figure S3A, Supporting Information). The transcriptional level of CsSH1 and CsSH2 in the fourth leaf was higher than that of the first, second, and third leaves (Figure S3D, Supporting Information). Furthermore, the expression of *CsSH1* and *CsSH2* can be readily induced by SA (Figure S3B, Supporting Information) and pathogen *Pcs* (Figure S3C, Supporting Information). Based on these findings, it is hypothesized that *CsSH1* and *CsSH2* may play a role in SA catabolism.

### 2.8 Identification of CsSH1 In Vitro and In Vivo

**Figure 5:**
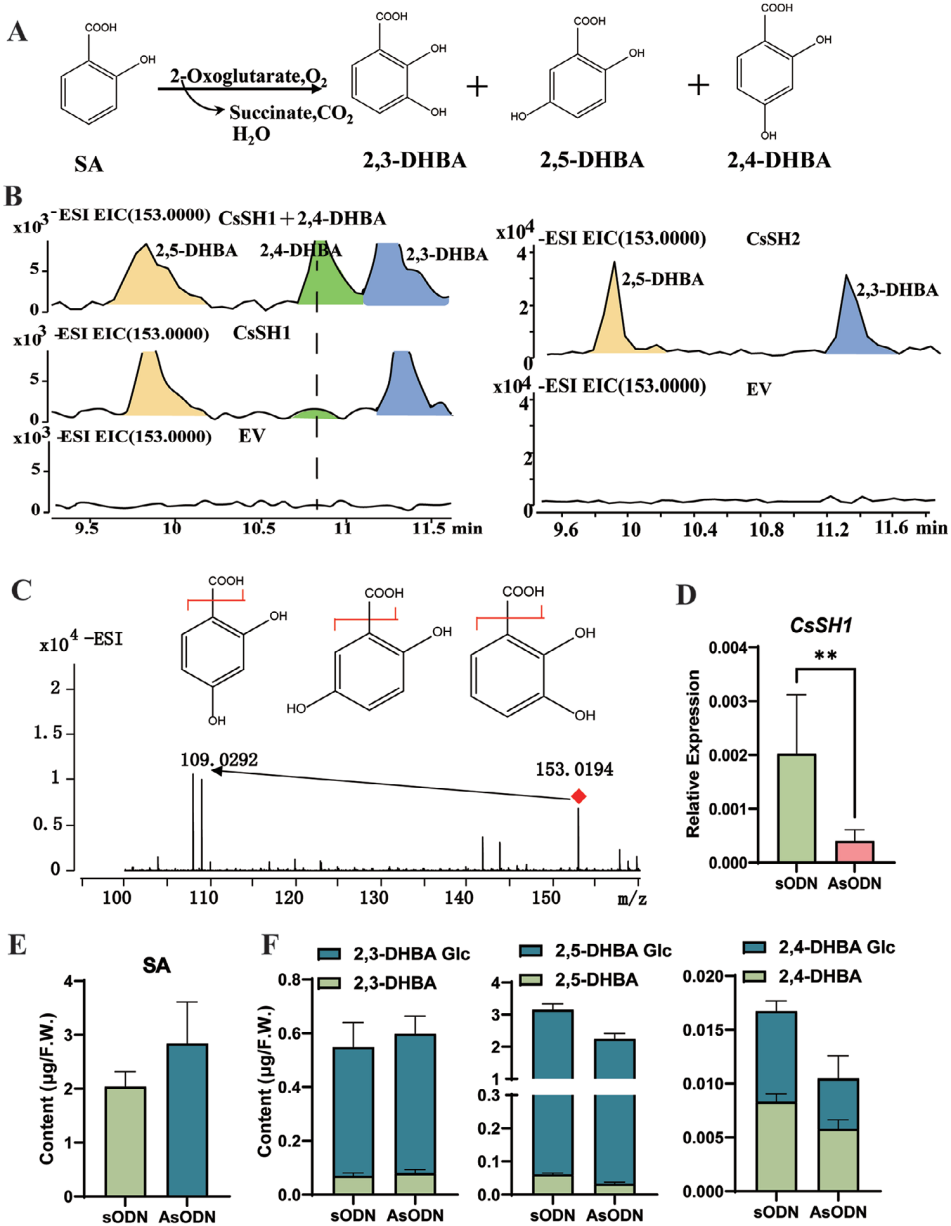
Identification of CsSH1 in Vitro and in Vivo. A) Biochemical reaction catalyzed by recombinant CsSH1 in vitro. B) HPLC profiles of 30‐min reaction of the recombinant CsSH1, CsSH2 protein and empty vector (EV) extracts incubated with SA. C) The CsSH1 enzymatic products to 2,3‐,2,4‐ and 2,5‐DHBA also have the same tandem ESI–mass spectra MS2, respectively. D) The relative expression levels of *CsSH1* in AsODN and sODN plants. E) Quantitative analysis of SA in AsODN and sODN plants. F) Quantitative analysis of DHBAs and glycosides in AsODN and sODN plants.

To validate this hypothesis, we independently expressed the recombinant CsSH1 and CsSH2 enzymes in BL21 (DE3) pLysS. Following established enzyme activity detection methods,^[^ ^7a^ ^]^ enzyme activity showed that both CsSH2 effectively utilized SA to produce 2,3‐DHBA and 2,5‐DHBA, confirming their 3‐ and 5‐hydroxylation activity (**Figure** **5**B). In addition to 2,3‐DHBA and 2,5‐DHBA, CsSH1 can also catalyze the formation of 2,4‐DHBA (Figure 5B). To investigate the role of CsSH1 in 2,4‐DHBA formation, we tried to suppress the expression of *CsSH1*. The expression analysis revealed the expression level of *CsSH1* was reduced to only 19% of that in control leaves (Figure 5D). As expected, the levels of both 2,4‐DHBA and 2,4‐DHBA glucoside reduced to 69% and 55%, respectively when compared to the control leaves (Figure 5F). Similarly reduced levels were also observed for both 2,5‐DHBA (54%) and its glycosylated form (71%). However, the levels of both2,3‐DHBA and 2,3‐DHBA glucoside were increased. Additionally, the level of SA content in *CsSH1*‐silenced tea leaves increased compared with that in the control plants (Figure 5E). These results indicate that CsSH1 plays a crucial role in converting SA into 2,4‐ DHBA and 2,5‐DHBA rather than 2,3‐DHBA in tea plants.

### 2.9 UGT95B17 Catalyzes the Formation of 2,4‐ and 2,5‐DHBA Glucosides In Vivo

**Figure 6:**
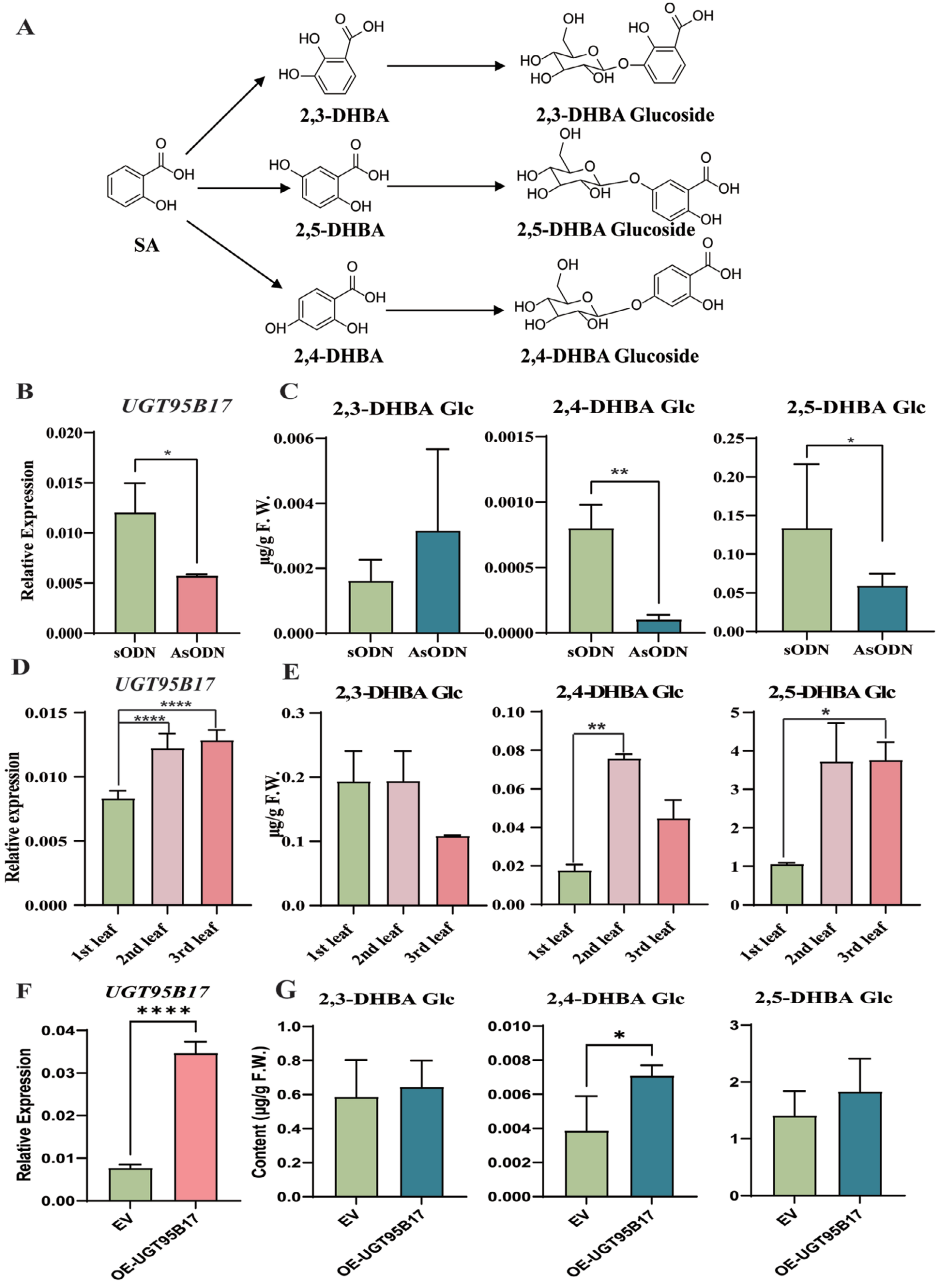
UGT95B17 catalyzed 2,4‐ and 2,5‐DHBA glucosides formation in vivo. A) Chemical structures of 2,3‐, 2,4‐, and 2,5‐DHBA glucoside. B) *UGT95B17* suppression (AsODN) and control (sODN) solution were injected into young leaves of tea plants. The RT‐qPCR results of *UGT95B17* suppression in tea plants at 24 h. C) Quantitative analysis of DHBA glucosides after suppression. D) The RT‐qPCR results of *UGT95B17* in leaves at three different stages of development (1st, First; 2nd, second; 3rd, third). E) DHBA glycosides concentrations in leaves at three different stages of development. F) The relative expression levels of *UGT95B17* in OE‐*UGT95B17* and EV plants G) Quantitative analysis DHBA glucosides in OE‐*UGT95B17* and EV plants.

To gain further insights into the role of UGT95B17 in DHBA glucosylation in the tea plant, its expression was transiently suppressed in tea leaves by the gene‐specific antisense oligodeoxynucleotide suppression (AsODN) strategy.^[^ ^15^ ^]^ Sense oligonucleotides (sODN) were used as negative control. The average transcript level of *UGT95B17* was significantly lower in leaves treated with AsODN‐*UGT95B17* than in control leaves (**Figure** **6**B).

The content of 2,3‐, 2,4‐ and 2,5‐DHBA glucosides in *UGT95B17‐*silenced leaves were detected by LC‐MS. Metabolite analyses revealed that *UGT95B17‐*silenced tea leaves produced significantly lower levels of 2,4‐ and 2,5‐DHBA glucosides when compared with the levels in control, whereas the content of 2,3‐DHBA glucoside was increased in *UGT95B17‐*silenced tea leaves (Figure 6B,C). In addition, we also determined *UGT95B17* expression and DHBA glucosides contents in the first, second and third leaves from the top of tea plants. The expression of *UGT95B17* was higher in the second and third leaves than in the first leaves (Figure 6D), which was consistent with the changes in the content of 2,4‐DHBA, 2,5‐DHBA glucosides (Figure 6E). On the contrary, the content of 2,3‐DHAB glucoside in the first and second leaves was higher than in the third leaves (Figure 6E). Our results indicated that UGT95B17 is involved in the formation of 2,4‐DHBA and 2,5‐DHBA glucosides *in planta*. Carbon metabolic flux was shifted to the 2,3‐DHBA glucoside when the formation pathway to the 2,4‐DHBA and 2,5‐DHBA glucosides was blocked, resulting in increased flux to 2,3‐DHBA glucoside in *UGT95B17‐*silenced tea leaves.

**Figure 7:**
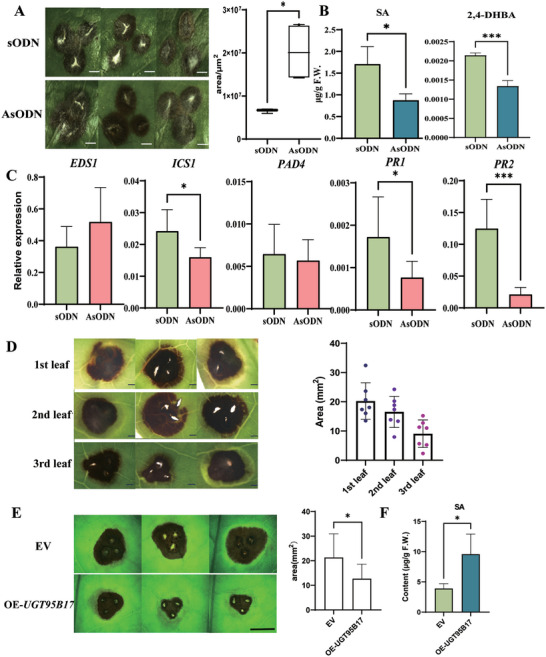
The pathogen phenotypes, 2,4‐DHBA and SA content, and related gene expression in *UGT95B17*‐silenced tea plants and OE‐*UGT95B17* tea plants. A) Disease symptoms after fungal infection in *UGT95B17* suppression (AsODN) and control (sODN) plants. Bar = 0.1 mm. B) Quantitative analysis of SA and 2,4‐DHBA after suppression of *UGT95B17*‐silenced and control tea leaves. C) The relative expression levels of SA biosynthesis and Pathogenesis‐related (PR) gene in sODN and AsODN tea plants. D) Pathogen symptoms after fungal infection were observed in three different leaves. Bar = 0.1 mm. E) Disease symptoms after fungal infection in OE‐*UGT95B17* and control (EV) plants. Bar = 0.5 mm. F) Quantitative analysis of SA in OE‐*UGT95B17* and EV plants.

To further investigate the function of UGT95B17, we tried to increase the expression of *UGT95B17* according to a previously established method.^[^ ^16^ ^]^ The relative expression level of *UGT95B17* was significantly up‐regulated in young leaves of tea plants compared to the control group (Figure 6F). Moreover, the content of SA in *UGT95B17* over‐expressed tea leaves was increased by 2.22‐fold compared to that in the control leaves (**Figure** **7**F). Similarly, the contents of 2,4‐, and 2,5‐DHBA glucoside were also increased by 1.84‐fold, and 1.39‐fold, respectively (Figure 6G). These findings suggest that over‐expression of *UGT95B17* in tea plants increased both SA and DHBA glucosides accumulation.

### 2.10 Suppression of *UGT95B17* Reduces Disease Resistance, Whereas Overexpression is Opposite in the Tea Plants

To further explore the role of UGT95B17 in disease resistance to *Pcs* in tea plants, the *UGT95B17*‐silenced and control tea leaves were inoculated with *Pcs*. On the fourth day after inoculation, the disease lesions were compared. The disease lesion areas in *UGT95B17*‐silenced tea leaves were significantly larger than that of the control leaves (Figure 7A) while the transcript levels of PR protein genes (*PR1* and *PR2*) were significantly decreased in *UGT95B17*‐silenced leaves compared with that in the control plants (Figure 7C). In addition, the SA and 2,4‐DHBA content, and the expression of *ICS1* (Isochorismate synthase 1) were decreased in *UGT95B17*‐silenced leaves (Figure 7B,C) while the transcript levels of *EDS1* (Enhanced disease susceptibility 1) and *PAD4* (Phytoalexin deficient 4) were not changed (Figure 7C). We transiently overexpressed *UGT95B17* in tea plants, followed by inoculation with *Pcs*. Our findings demonstrated that *UGT95B17* over‐expressed tea leaves exhibited a reduced lesion size and enhanced resistance to *Pcs* compared to the control leaves (Figure 7E).

Moreover, we examined *UGT95B17* expression at different leaf positions in tea plants. The expression of *UGT95B17* in the second and third leaves was higher than that in the first leaves (Figure 6D). The first, second and third leaves were inoculated with *Pcs*. Four days after inoculation, the size of the lesion areas in the second and third leaves was smaller than that in the first leaves (Figure 7D). These results suggest that UGT95B17‐mediated 2,4‐ and 2,5‐glucosides formation influences SA homeostasis and disease resistance during pathogen infection.

### 2.11 Exogenous Application of 2,4‐DHBA Enhances Disease Resistance of Tea Plants

**Figure 8:**
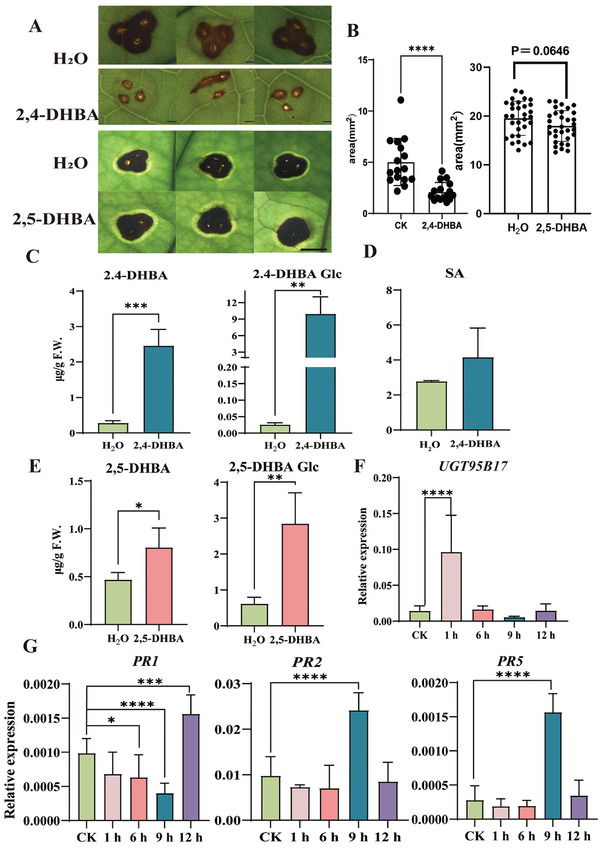
The pathogen symptoms, DHBA glucosides content and related gene expression after exogenous application of 2,4‐DHBA. A) Pathogen symptoms after fungal infection by water, exogenous 2,4‐DHBA (Bar = 0.1 mm), 2,5‐DHBA (Bar = 0.5 mm). B) The average lesion statistics chart at 4 dpi. CK stands for contrast in water treatment. C) Quantitative analysis of 2,4‐DHBA and its glycoside after exogenous 2,4‐DHBA. D) Quantitative analysis of SA after exogenous 2,4‐DHBA. E) Quantitative analysis of 2,5‐DHBA and its glucoside after exogenous 2,5‐DHBA. F) The RT‐qPCR results of *UGT95B17* after exogenous 2,4‐DHBA. CK represents the control group in the water treatment. G) The RT‐qPCR results of PR genes after exogenous 2,4‐DHBA and water treatment (CK).

To verify the role of 2,4‐DHBA and 2,5‐DHBA in disease resistance, 2 mm 2,4‐DHBA and 2,5‐DHBA was applied to tea leaves for 24 h, and then the leaves were inoculated with a spore suspension of *Pcs*. After exogenous supplement of 2,4‐DHBA, the expression level of *UGT95B17*, *PR1*, *PR2* and *PR5* was significantly induced at 1, 12, 9, and 9 h, respectively (**Figure** **8**F,G), and the content of 2,4‐DHBA and its glucoside content was increased, indicating that tea plants can absorb exogenous 2,4‐DHBA and transform it to its glucoside in tea plants (Figure 8C). The SA content was slightly increased after 2,4‐DHBA application compared with the control but did not reach the statistically significant level (Figure 8D). The leaf lesion areas after 2,4‐DHBA exposure were significantly smaller than that of control tea plants (Figure 8A,B; Figure S4, Supporting Information), suggesting that exogenous application of 2,4‐DHBA could enhance plant resistance to *Pcs*.

The content of 2,5‐DHBA and its glucoside also increased after application of 2,5‐DHBA (Figure 8E). However, compared with the water control, there was no difference in lesion area of the tea leaves treated with 2,5‐DHBA (Figure 8A,B), indicating that the higher level of 2,5‐DHBA and its glucoside could not increase plant resistance to *Pcs*. It seems that 2,4‐DHBA plays a key role in disease resistance of tea plants.

### 2.12 UGT95B17 Positively Regulates Disease Resistance via Effecting 2, 4‐DHBA Rather than SA Homeostasis in Tea Plants

**Figure 9:**
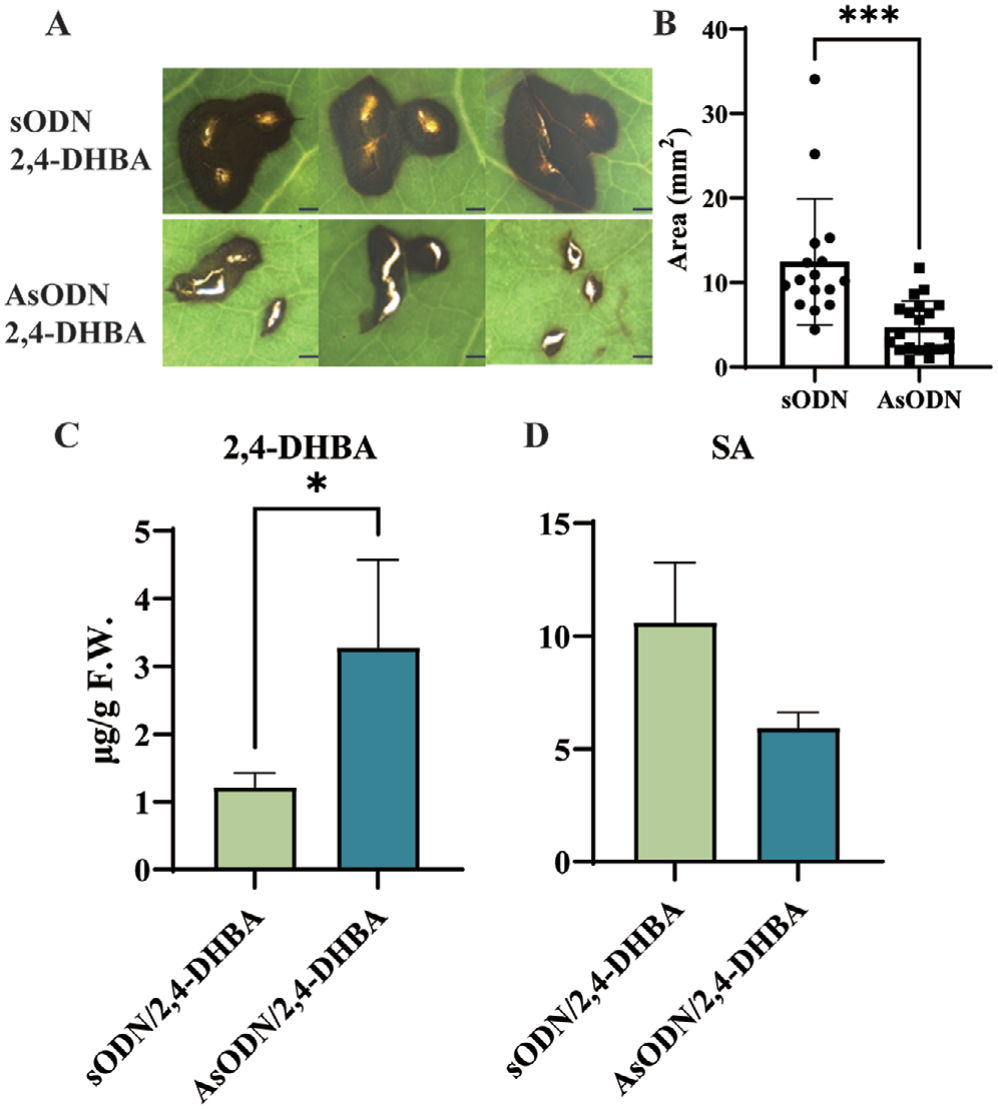
Exogenous application of 2,4‐DHBA effect pathogen phenotype, 2,4‐DHBA but not SA content in *UGT95B17*‐silenced tea plants. A) Disease symptoms after fungal infection were observed by suppression gene and then exogenous 2,4‐DHBA. B) Quantitative analysis of 2,4‐DHBA. C) Quantitative analysis of SA. Bar = 0.1 mm.

To further explore the role of *UGT95B17*‐mediated 2,4‐DHBA homeostasis in disease resistance, we applied exogenous 2,4‐DHBA to the *UGT95B17*‐silenced and control tea leaves. After inoculated with *Pcs* for 4 days, the phenotypes were analyzed. The lesion size was smaller in the *UGT95B17*‐silenced tea leaves than in the controls (**Figure** **9**A,B). Compared with control leaves, there was a significant increase in 2,4‐DHBA content (Figure 9C), however, the level of SA decreased significantly (Figure 9D). All in all, these results suggested that UGT95B17 positively regulates disease resistance by affecting 2,4‐DHBA rather than SA homeostasis in tea plants.

## 3 Discussion

### 3.1 Discovery of 2,4‐DHBA Glucoside as Native Potentially Bioactive Molecule in Plants

**Figure 10:**
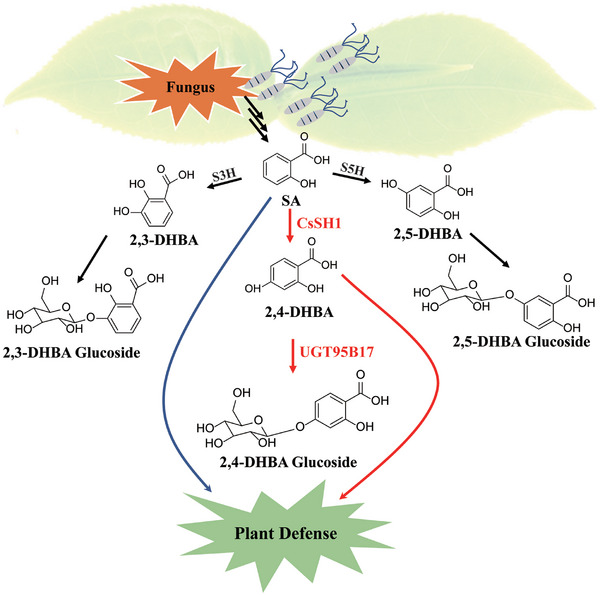
Proposed working model for the regulation of 2,4‐DHBA homeostasis and UGT95B17‐mediated pathogen defense in tea plants. The S3H and S5H catalyze the SA to form 2,3‐DHBA and 2,5‐DHBA that will be subsequently conjugated by sugars to produce 2,3‐DHBA and 2,5‐DHBA sugar conjugates. The SA can be converted to 2,4‐DHBA by CsSH1 that is then converted to its sugar conjugates by UGT95B17. When the tea plant is infected by a fungus, *UGT95B17* expression is up‐regulated, leading to the accumulation of 2,4‐DHBA and its glucoside. 2,4‐DHBA application induces the expression of PR genes and the accumulation of SA content. As a consequence, levels of PR proteins increase, and plant defense is activated.

In plants, SA may undergo biologically relevant chemical modifications such as glycosylation, methylation, amino acid conjugation, and hydroxylation. Although previous studies have confirmed that 2,3‐DHBA and 2,5‐DHBA, usually in glycosylated form, are hydroxylated products of SA,^[^ ^8^, ^17^ ^]^ the mode of action of SA‐related metabolites remains unclear. Some UGTs have been identified that are capable of catalyzing the transformation of 2,4‐DHBA in vitro,^[^ ^11^ ^]^ but it is unknown whether 2,4‐DHBA and its glucose conjugate are present in plants. In this study, we have identified for the first time that 2,4‐DHBA is an endogenously produced plant metabolite that plays a critical role in SA catabolism and homeostasis in response to plant diseases (**Figure** **10**). 2,4‐DHBA and its glucoside were rarely accumulated in unstressed tea plants but strongly induced in leaves of *Pcs*‐challenged tea plants (Figure 1C). In *Arabidopsis*, 2,5‐DHBA and 2,3‐DHBA are the major metabolic forms of SA and their content can be significantly induced by pathogens.^[^ ^17^, ^18^ ^]^ However, in tea plants, in additional to 2,5‐DHBA and 2,3‐DHBA, 2,4‐DHBA was also detected as hydroxylated SA, suggesting a novel SA metabolic pathway in plants. We observed the metabolism of 2,4‐DHBA in tea plants by administering the ^13^C_6_‐SA isotope, whereas the specific enzyme responsible for its production remains unclear. Here, we also identified CsSH1 as a hydroxylase present in tea plants that synthesizes 2,4‐DHBA both in vivo and in vitro (Figure 5). Previous studies have reported the identification of SA 3‐hydroxylases (S3H) and 5‐hydroxylases (S5H) in *Arabidopsis*.^[^ ^7^ ^]^ Similar to *S3H* and *S5H*, we found that the expression of *CsSH1* can be induced by SA and pathogen *Pcs* (Figure S3, Supporting Information).

The response of plants to pathogen includes the increase of SA and the activation of PR gene expression.^[^ ^19^ ^]^ The exact roles of 2,3‐DHBA and 2,5‐DHBA in *Arabidopsis* are not known, but 2,5‐DHBA is thought to play a signaling role in the activation of inducible defenses. The signaling role of 2,5‐DHBA is complementary to SA, because the exogenous application of 2,5‐DHBA induces the formation of a different set of PR proteins.^[^ ^9b,c^ ^]^ In our study, we showed that exogenous application of 2,5‐DHBA did not increase disease resistance in tea plants. However, application of 2,4‐DHBA could induce the expression of PR genes and thus increase resistance to *Pcs* in tea plants (Figure 8), indicating that 2,4‐DHBA might be function as a potentially bioactive molecule in tea plants.

### 3.2 *UGT95B17* is Involved in 2,4‐DHBA Glucoside Formation, Revealing Complex SA Metabolic Pathway in Plants

Studies already suggested that glycosylation of DHBA may be involved in plant immunity. Several glycosyltransferases capable of catalyzing the transformation of 2,3‐DHBA and 2,5‐DHBA have been identified UGT89A2 was identified as a 2,5‐DHBA xylosyltransferase in *Arabidopsis*,^[^ ^10b^ ^]^ whereas UGT76D1 glycosylates both 2,3‐DHBA and 2,5‐DHBA using either UDP‐Glc or UDP‐Xyl as sugar donors.^[^ ^10a^ ^]^ Besides, UGT75B1 forms a glucose ester with the carboxyl group of BA, 3‐HBA, 4‐HBA, and 3,4‐DHBA.^[^ ^11^ ^]^ In addition to 2,3‐DHBA and 2,5‐DHBA, 2,4‐DHBA and its glucoside were unambiguously discovered as novel SA derivative in tea plants in our current study. The enzyme responsible for the formation of 2,4‐DHBA and its glycosides was previously unknown in plants.

In this study, UGT95B17 was identified as a novel DHBA glucosyltransferase in tea plants, and it can catalyze the glucosylation of 2,4‐ and 2,5‐DHBA (Figures 3E,F and 6C,G). *UGT95B17*‐silenced tea leaves contained much lower concentrations of 2,4 and 2,5‐DHBA glucosides (Figure 6B,C), whereas the levels were elevated in the overexpressing tea plants (Figure 6F,G). The down‐regulation of UGT95B17 gene expression resulted in a reduction in the content of 2,4‐DHBA and its glucoside. This suggests that the down‐regulation of *UGT95B17* leads to decreased 2,4‐DHBA via feedback inhibition of the potential structural genes.^[^ ^20^ ^]^ However, further investigation is required to pursue and understand this regulatory mechanism in detail. Although phylogenies can be used to predict the functions of unknown genes, some UGTs that cluster together may have very different functions. *UGT7*, *UGT95A1*, and *UGT95B7* belong to the same group, but these enzymes catalyze the glycosylation of flavonoids.^[^ ^21^ ^]^ Here, it was shown that *UGT95B17*, which is a member of the new UGT95B subfamily, exhibits specific glycosylation activity toward DHBA, and is structurally distinct from *UGT89A2* and *UGT76D1* (Figure 3B). Our results revealed that UGT95B17 is a novel DHBA glycosyltransferase responsible for the formation of 2,4‐ and 2,5‐DHBA glucosides in tea plants.

### 3.3 UGT95B17 Positively Regulates Disease Resistance by Affecting 2,4‐DHBA Rather than SA Homeostasis in Tea Plants

SA is a crucial signal for plant defense responses. Its levels and changes in tissues directly influence the SA‐dependent defense responses of plants. Glycosylation inactivates SA and allows vacuolar storage of relatively large quantities of SA due to reduced toxicity. SA has been shown to be converted to SAG and SGE. SAG is thought to be a more stable storage form of SA, and its levels have been shown to increase in parallel with free SA in plant defense responses.^[^ ^22^ ^]^ Very recently, we found that the UGT87E7 ‐mediated salicylic acid glucose ester formation plays a positive role in plant disease resistance by modulating salicylic acid homeostasis,^[^ ^4b^ ^]^ suggesting the importance of downstream SA metabolic processes in SA signaling.

It is likely that DHBA glycosides have important roles in plant defense response. The 2,5‐DHBA glucosides accumulated in tomato and cucumber plants after infection by different pathogens,^[^ ^9^, ^23^ ^]^ while 2,3‐DHBA xylosides increased in *Arabidopsis* after induction by *PstDC3000*.^[^ ^8^ ^]^ A very recent study showed that UGT76D1‐medated 2,3‐DHBA and 2,5‐DHBA glycosylation plays a key role in the plant innate immune response through modulating SA homeostasis.^[^ ^10a^ ^]^ In our current study, *UGT95B17* expression was induced by *Pcs* and SA. The expression levels of PR1 and PR2 genes were reduced in *UGT95B17*‐silenced tea plants, leading to more severe disease symptoms (Figure 7A,C). This phenomenon is similar to that of UGT76D1 knockout mutants of *Arabidopsis*, which showed a delayed immune response, with reduced levels of DHBA glycosides.^[^ ^10a^ ^]^ However, the increase in 2,4‐DHBA glucoside and 2,5‐DHBA glucoside showed that 2,4‐DHBA glucosylation plays an important role in the immune responses of tea plant to *Pcs* (Figure 8). Furthermore, exogenous application of 2,4‐DHBA to *UGT95B17*‐silenced tea leaves reduced the lesion size caused by *Pcs* in comparison to control tea leaves (Figure 9A,B). Compared with control leaves, there was a significant increase in 2,4‐DHBA content (Figure 9C), but SA content decreased (Figure 9D). Based on these findings, UGT95B17 positively regulates disease resistance by affecting 2,4‐DHBA rather than SA homeostasis in tea plants. Additionally, 2,4‐DHBA glucoside seems like a storage form of active 2,4‐DHBA, which requires further investigation. Furthermore, UGT95B17 appears to play a role in regulating the content of 2,4‐DHBA. In most cases of plant hormones, glycosylation of plant hormone will reduce the activity of them. For example, ABA glycosylation catalyzed by UGT71C5 results in the formation of ABA‐GE, an inactive ester derivative of ABA.^[^ ^24^ ^]^ The UGT84B1 is responsible for the production of IAA‐β‐d‐glucoside and participates in the deactivation of IAA.^[^ ^25^ ^]^ Therefore, we speculate that glycosylation of 2,4‐DHBA plays an important role in plant immunity by regulating the content of 2,4‐DHBA. However, further investigations are necessary to study the accurate role of UGT95B17‐associated 2,4‐DHBA glucosylation in plant immunity.

From the results, we derive the following hypothesis (Figure 10). After fungal attack, SA is produced and is hydroxylated to form 2,3‐, 2,4‐ and 2,5‐DHBA. CsSH1 as a salicylic acid 4‐hydroxylase that catalyzes the formation of 2,4‐DHBA by hydroxylating SA. These DHBAs are subsequently conjugated to sugars to produce the sugar conjugates. When the tea plant is infected by a fungus, the expression of *UGT95B17* is induced, and the contents of DHBA, especially 2,4‐DHBA and its glucosides increase. In contrast, when the expression of *UGT95B17* is suppressed, 2,4‐DHBA glycoside formation is blocked, and the level of SA content decreases via an unknown positive feedback loop. Exogenous application of 2,4‐DHBA to *UGT95B17*‐silenced tea leaves results in an increase in 2,4‐DHBA, a decrease in SA, and increased disease resistance. Overall, our results demonstrate that 2,4‐DHBA glucosylation mediated by UGT95B17 plays a positive role in disease resistance in tea plants and highlight the role of 2,4‐DHBA as a potentially bioactive molecule in the establishment of basal resistance.

## 4 Experimental Section

### Plants and Pathogenic Fungus

Tea samples (*Camellia sinensis* cultivar Shuchazao) were used as study materials. Two years old tea seedlings were collected from the horticultural research station of Anhui Agricultural University during the early spring. Plant material of experiment was frozen in liquid nitrogen and stored in −80 °C until use *Pseudopestalotiopsis camelliae‐sinensis* (*Pcs*) isolated from tea grey blight.^[^ ^4b^ ^]^ *Pcs* was cultivated in PDA medium at 28 °C for 2–3 days.

### Pathogen Infection Experiment

Spores of *Pcs* from the culture were suspended in sterile distilled water and diluted the spore suspension to a concentration of 10^7^ per mL.^[^ ^26^ ^]^ Pathogen infection experiments were based on Hu's method.^[^ ^4b^ ^]^ First, the leaves of tea were sterilized with 75% alcohol and washed two or three times with sterilized water. Acupuncture method was used to inoculate *Pcs* by 50 µL spore suspension, distilled water as control. The treated tea seedlings were bagged and cultured in the greenhouse. The leaves were collected at 1, 4, 7, and 10 days after inoculation for subsequent transcription and metabolism. The infection genotypes were taken as photographs. For each genotype after infection, at least ten leaves from different plants were evaluated in each experiment.

### Suppression of *UGT95B17* Expression in Tea Plants

Candidate antisense oligonucleotides (AsODN) were selected using SOLIGO software ^[^ ^27^ ^]^ with *UGT95B17* as the input sequence (Table S3, Supporting Information). AsODNs were synthesized by the General Biosystems Company (Anhui, China). To silence *UGT95B17* in leaves that were still attached to the plant, 1 mL of 60 µm AsODN UGT95B17 solution was injected into tea seedlings, and seedlings injected with the sODN were used as controls. After 24 h of incubation, the leaves were inoculated as described below.

### RNA Isolation, cDNA Cloning, and Sequence Analysis

According to the manufacturer's instructions, total RNA was isolated using RNAisomate for Plant Tissue (Takara, Dalian, China) and RNAiso Plus (Takara, Dalian, China). The cDNA was synthesized by reverse transcription from the total RNA using PrimeScript RTMaster Mix (Takara, DaLian, China). The open reading frame sequence was amplified using Phusion High‐Fidelity DNA Polymerase (NewEngland Biolabs, MA, USA), and the PCR products were purified with a MiniBEST Agarose Gel Extraction Kit (Takara, Dalian, China) and ligated into the PGEX‐4T1 simple vector and subsequently transformed into TransT1 competent cells. Wild type tea plant tissues of different developmental stages (first leaf, second leaf, and third leaf), were harvested, and nucleic acid was isolated by the same method.

### Quantitative Real Time PCR Analysis

According to the reported method, qRT‐PCR was performed in this study.^[^ ^28^ ^]^ The gene specific primer sequences are provided in Table S3 (Supporting Information). The glyceraldehyde‐3‐phosphate dehydrogenase (GAPDH) gene was used as an internal reference gene, and the relative expression was calculated using the 2^–ΔCT^ method.^[^ ^29^ ^]^

### Expression Vector pGEX‐4T1‐UGT95B17

The full‐length UGT95B17 was amplified by PCR from *C. sinensis* var. *sinensis*. “Shuchazao” (primers as shown in Table S3, Supporting Information). According to a published protocol,^[^ ^30^ ^]^ the amplified full‐length sequences were digested with BamH1 and Smal1, which were cloned into the expression vector pGEX‐4T‐1 (Amersham Biosciences, Freiburg, Germany). And then the recombinant plasmids were transferred into Trans1‐T1‐competent cells.

### Heterologous Protein Expression and Purification

The recombinant plasmids were transformed into *E. coli* strain BL21(DE3) pLysS cells, which were cultured overnight at 37 °C in Luria–Bertani (LB) liquid medium containing ampicillin (50 µg mL^−1^) and chloramphenicol (50 µg mL^−1^). the culture was diluted and grown until the optical density (OD_600_) of the cultured cells reached 0.6–0.8. Heterologous protein purification method was drawn on the reported method.^[^ ^31^ ^]^

### Enzymatic Activity Assay

In the initial screening, each reaction mixture (25 µL in total) contained Tris‐HCl buffer (50 mm, pH 7.5, 10% glycerol, and 10 mm 2‐mercaptoethanol), 250 mm UDP‐glucose, alcohol substrates (0.4 µL of a 10 mm stock solution), and purified protein (0.5–1 µg per reaction) according to Jing et al.^[^ ^30^ ^]^ The reaction mixture was incubated at 30 °C for 30 min and was stopped by mixing the reaction solution with the same volume of UDP‐GloTM assay reagent.^[^ ^32^ ^]^ Optimum reaction temperature and pH were determined.^[^ ^30^ ^]^ The optimized conditions were used for determination of the kinetic parameters, and at least seven different substrate concentrations were used.

The enzyme assay was performed according to the previously described method.^[^ ^7a^ ^]^ The reaction mixture (100 µL) contained 5 mm DTT, 4 mm sodium ascorbate, 1 mm 2‐oxopentanedioic acid, 0.4 mm FeSO4, 0.1 mg mL^−1^ hydrogen peroxide, 50 mm Tris‐HCl (pH 8) or other buffer, 15 mg recombinant protein, and 0.4 mm SA. All reactions were initiated by the addition of the enzyme and stopped by adding 100 mL of 50% (v/v) acetonitrile, followed by 1 min heating in boiling water to denature the proteins. After centrifugation at maximum speed for 10 min, the supernatant was analyzed by LC‐MS.

### Transient Overexpression of *UGT95B17* in Leaves of Tea Plants

A bud and two leaves of tea plants transiently overexpressing *UGT95B17* were obtained using a previously described procedure with minor modifications.^[^ ^16^ ^]^ Agrobacterium transformed with the *UGT95B17* construct (GV3101 (pSoup‐p19) Chemically Competent Cell) was grown in liquid LB medium and then resuspended to a final OD of 1.0 for injection. Agrobacterium carrying the empty pCAMBIA 1302 vector with eGFP was injected as a control. To minimize the effect of mechanical damage to the leaves, injections were made into petioles. Each experiment consisted of at least six replicates. Each replicate contained one bud and two leaves in 15 replicates. Samples were collected 2–3 days after injection, immediately frozen in liquid nitrogen, ground, mixed evenly, and divided into several parts for qRT‐PCR analysis, disease resistance detection, and metabolite detection.

### Identification of Products by LC‐MS

Standard assays contained 5 mm UDP‐glucose, 200 µm substrate, and *UGT95B17* protein (15 to 20 µg total protein) in a volume of 200 µL. Enzyme assays were incubated at 30 °C overnight. The reaction solutions were extracted twice with 200 µL ethyl acetate, and the organic solvent was vaporized. The residue was dissolved in 50 µL methanol/water (1:1, vol) and analyzed by LC–MS. The LC system selected for this purpose was a DIONEX Ultimate 3000 UHPLC system (Thermo Fisher Scientific, Waltham, MA, USA) with an auto‐sampler. HPLC was performed on a reverse‐phase C18 column (1.8 µm, 100 × 2.1 mm) with a solvent flow rate of 0.2 mL min^−1^ at acolumn temperature of 40 °C. The sample injection volume was set at 1 µL. The mobile phases were constituted with solvent A, water mixed with 0.1% formic acid (v/v), and solvent B, acetonitrile containing 0.1% formic acid (v/v). The solvent gradient adopted was as follows: 0 min 5% B, 0–2 min 5% B, 2–22 min 45% B, 22–26 min 65% B, 26–28 min 65% B, 28–29 min 5% B, 29–30 min 5% B. Products were identified by comparison of their UV, MS, and MS2 spectra with those from literature and of commercially available reference material.

### Stable Isotope Labeling Experiments

To investigate whether 2,4‐DHBA was a SA derivative, tea branches collected were fed with SA‐^13^C_6_(600 µL, 300 µm) for 24 h. The stable isotope tracing experiment was completed using four biological replicates. The isotope‐labeled products were determined as described in the “Identification of products by LC‐MS” section.

### Phylogenetic Tree Analysis

The phylogenetic tree was constructed by the neighbor‐joining method ^[^ ^30^ ^]^ using MAGA11 software based on the deduced amino acid sequences of UGTs. The 0.1 scale bar indicates 0.1 nucleotide substitutions per site.

### Statistical Analysis

All experiments were carried out with at least three independent biological replicates. Each measurement was carried out in triplicate. Data were presented as mean ± SD. Data were statistically analyzed by one‐way ANOVA performed using SPSS. Asterisks indicate significant differences relative to the wild type or control (^*^ *p* < 0.05, ^**^ *p* < 0.01).

## Conflict of Interest

The authors declare no conflict of interest.

## Author Contributions

M.L. and C.S. designed the research, M.L., Y.Z., Y.F, X.T., W.Z., K.Y., and Y.T., performed the research, M.L., Q.W., J.C., M.Z., J.J., J.W., and M.Z., contributed new analytic and computational tools, M.L., and C.S. analyzed data, M.L., W.S., and C.S. wrote the paper. All authors agreed to the list of authors and the identified contributions of those authors. The author responsible for distribution of materials integral to the findings presented in this article in accordance with the policy described in the Instructions for Authors is Chuankui Song (cksong@ahau.edu.cn).

## Supporting information

Supporting Information

## Data Availability

The data that support the findings of this study are available from the corresponding author upon reasonable request.

## References

[1] X. Li , H. Lin , W. Zhang , Y. Zou , J. Zhang , X. Tang , J.‐M. Zhou , Proc. Natl. Acad. Sci. USA 2005, 102, 12990. 16123135 10.1073/pnas.0502425102 PMC1200263

[2] a) M. Yuan , B. P. M. Ngou , P. Ding , X.‐F. Xin , Curr. Opin. Plant Biol. 2021, 62, 102030,; 33684883 10.1016/j.pbi.2021.102030

[3] a) A. C. Vlot , D. A. Dempsey , D. F. Klessig , Annu. Rev. Phytopathol. 2009, 47, 177; 19400653 10.1146/annurev.phyto.050908.135202

[4] a) A. M. George Thompson , C. V. Iancu , K. E. Neet , J. V. Dean , J.‐Y. Choe , Sci. Rep. 2017, 7, 46629; 28425481 10.1038/srep46629 PMC5397973

[5] P.‐P. Liu , C. C. Von Dahl , S.‐W. Park , D. F. Klessig , Plant Physiol. 2011, 155, 1762. 21311035 10.1104/pp.110.171694 PMC3091099

[6] a) Y. Chen , H. Shen , M. Wang , Q. Li , Z. He , Acta Biochim. Biophys. Sin. 2013, 45, 827; 23842113 10.1093/abbs/gmt078

[7] a) K. Zhang , R. Halitschke , C. Yin , C.‐J. Liu , S.‐S. Gan , Proc. Natl. Acad. Sci. USA 2013, 110, 14807; 23959884 10.1073/pnas.1302702110 PMC3767541

[8] M. Bartsch , P. Bednarek , P. D. Vivancos , B. Schneider , E. Von Roepenack‐Lahaye , C. H. Foyer , E. Kombrink , D. Scheel , J. E. Parker , J. Biol. Chem. 2010, 285, 25654. 20538606 10.1074/jbc.M109.092569 PMC2919129

[9] a) J. M. Bellés , R. Garro , J. Fayos , P. Navarro , J. Primo , V. Conejero , Mol. Plant‐Microbe Interact. 1999, 12, 227; 10.1094/MPMI-20-11-1439 17977155

[10] a) X.‐X. Huang , G.‐Q. Zhu , Q. Liu , L. Chen , Y.‐J. Li , B.‐K. Hou , Plant Physiol. 2018, 176, 3103; 29483147 10.1104/pp.17.01530 PMC5884596

[11] E.‐K. Lim , C. J. Doucet , Y. Li , L. Elias , D. Worrall , S. P. Spencer , J. Ross , D. J. Bowles , J. Biol. Chem. 2002, 277, 586. 11641410 10.1074/jbc.M109287200

[12] a) Z. H. Wang , Z. X. Zhao , N. Hong , D. Ni , L. Cai , W. X. Xu , Y. N. Xiao , Plant Dis 2017, 101, 1802; 30676920 10.1094/PDIS-04-17-0495-RE

[13] H.‐Y. Chen , X. Li , Plant J. 2017, 89, 195. 27411741 10.1111/tpj.13271

[14] P. I. Mackenzie , I. S. Owens , B. Burchell , K. W. Bock , A. Bairoch , A. Belanger , S. F. Gigleux , M. Green , D. W. Hum , T. Iyanagi , D. Lancet , P. Louisot , J. Magdalou , J. Roy Chowdhury , J. K. Ritter , T. R. Tephly , H. Schachter , T. Tephly , K. F. Tipton , D. W. Nebert , Pharmacogenetics 1997, 7, 255. 9295054 10.1097/00008571-199708000-00001

[15] M. Zhao , J. Jin , T. Gao , N. Zhang , T. Jing , J. Wang , Q. Ban , W. Schwab , C. Song , Front. Plant Sci. 2019, 10, 1675. 31929783 10.3389/fpls.2019.01675 PMC6941654

[16] Y. Chen , C. Jiang , S. Yin , J. Zhuang , Y. Zhao , L. Zhang , X. Jiang , Y. Liu , L. Gao , T. Xia , The Plant Journal 2022, 113, 576. 36534122 10.1111/tpj.16069

[17] R. K. Ibrahim , G. H. N. Towers , Nature 1959, 184, 1803.

[18] D.'. A. Dempsey , A. C. Vlot , M. C. Wildermuth , D. F. Klessig , Arabidopsis Book 2011, 9, e0156. 22303280 10.1199/tab.0156 PMC3268552

[19] a) Y. Yang , J. Shah , D. F. Klessig , Genes Dev. 1997, 11, 1621; 9224713 10.1101/gad.11.13.1621

[20] R. Yin , B. Messner , T. Faus‐Kessler , T. Hoffmann , W. Schwab , M.‐R. Hajirezaei , V. Von Saint Paul , W. Heller , A. R. Schäffner , J. Exp. Bot. 2012, 63, 2465. 22249996 10.1093/jxb/err416 PMC3346215

[21] a) S. Witte , S. Moco , J. Vervoort , U. Matern , S. Martens , Planta 2009, 229, 1135; 19238428 10.1007/s00425-009-0902-x

[22] a) A. J. Enyedi , N. Yalpani , P. Silverman , I. Raskin , Proc. Natl. Acad. Sci. USA 1992, 89, 2480; 1549613 10.1073/pnas.89.6.2480 PMC48682

[23] J. Fayos , J. M. Bellés , M. P. López‐Gresa , J. Primo , V. Conejero , Phytochemistry 2006, 67, 142. 16321412 10.1016/j.phytochem.2005.10.014

[24] Z. Liu , J.‐P. Yan , D.‐K. Li , Q. Luo , Q. Yan , Z.‐B. Liu , L.‐M. Ye , J.‐M. Wang , X.‐F. Li , Y. Yang , Plant Physiol. 2015, 167, 1659. 25713337 10.1104/pp.15.00053 PMC4378179

[25] K.‐I. Hayashi , K. Arai , Y. Aoi , Y. Tanaka , H. Hira , R. Guo , Y. Hu , C. Ge , Y. Zhao , H. Kasahara , K. Fukui , Nat. Commun. 2021, 12, 6752. 34811366 10.1038/s41467-021-27020-1 PMC8608799

[26] D. O. C. Harteveld , O. A. Akinsanmi , A. Drenth , Eur. J. Plant Pathol. 2014, 139, 789.

[27] Y. Ding , Nucleic Acids Res. 2003, 31, 7280. 14654704 10.1093/nar/gkg938 PMC297010

[28] C. Song , L. Gu , J. Liu , S. Zhao , X. Hong , K. Schulenburg , W. Schwab , Plant Cell Physiol. 2015, 56, 2478. 26454881 10.1093/pcp/pcv151

[29] M. Lu , J. Han , B. Zhu , H. Jia , T. Yang , R. Wang , W.‐W. Deng , Z.‐Z. Zhang , Planta 2019, 249, 363. 30209617 10.1007/s00425-018-3007-6

[30] T. Jing , N. Zhang , T. Gao , M. Zhao , J. Jin , Y. Chen , M. Xu , X. Wan , W. Schwab , C. Song , Plant, Cell Environ 2019, 42, 1352. 30421786 10.1111/pce.13479

[31] Y. Chen , X. Guo , T. Gao , N. Zhang , X. Wan , W. Schwab , C. Song , Hortic. Res. 2020, 7, 25. 32140234 10.1038/s41438-020-0248-x PMC7049299

[32] M. O. Sheikh , S. M. Halmo , S. Patel , D. Middleton , H. Takeuchi , C. M. Schafer , C. M. West , R. S. Haltiwanger , F. Y. Avci , K. W. Moremen , L. Wells , Glycobiology 2017, 27, 206. 28177478 10.1093/glycob/cww114 PMC5789813

