# Supplementary material for: 2,4‐Dihydroxybenzoic Acid, a Novel SA Derivative, Controls Plant Immunity via UGT95B17‐Mediated Glucosylation: A Case Study in Camellia Sinensis
Source: Adv Sci (Weinh). 2023 Dec 8;11(7):2307051. doi: 10.1002/advs.202307051 (PMC10870048; doi:10.1002/advs.202307051)
Supplement: Supplementary file 1 — Supporting Information [file ADVS-11-2307051-s001.pdf]

ADVANCED SCIENCE
Open Access

Supporting Information

for *Adv. Sci.*, DOI 10.1002/advs.202307051

2,4-Dihydroxybenzoic Acid, a Novel SA Derivative, Controls Plant Immunity via UGT95B17-Mediated Glucosylation: A Case Study in *Camellia Sinensis*

*Mengqian Lu, Yifan Zhao, Yingying Feng, Xiaoyan Tang, Wei Zhao, Keke Yu, Yuting Pan, Qiang Wang, Jilai Cui, Mengting Zhang, Jieyang Jin, Jingming Wang, Mingyue Zhao, Wilfried Schwab and Chuankui Song**

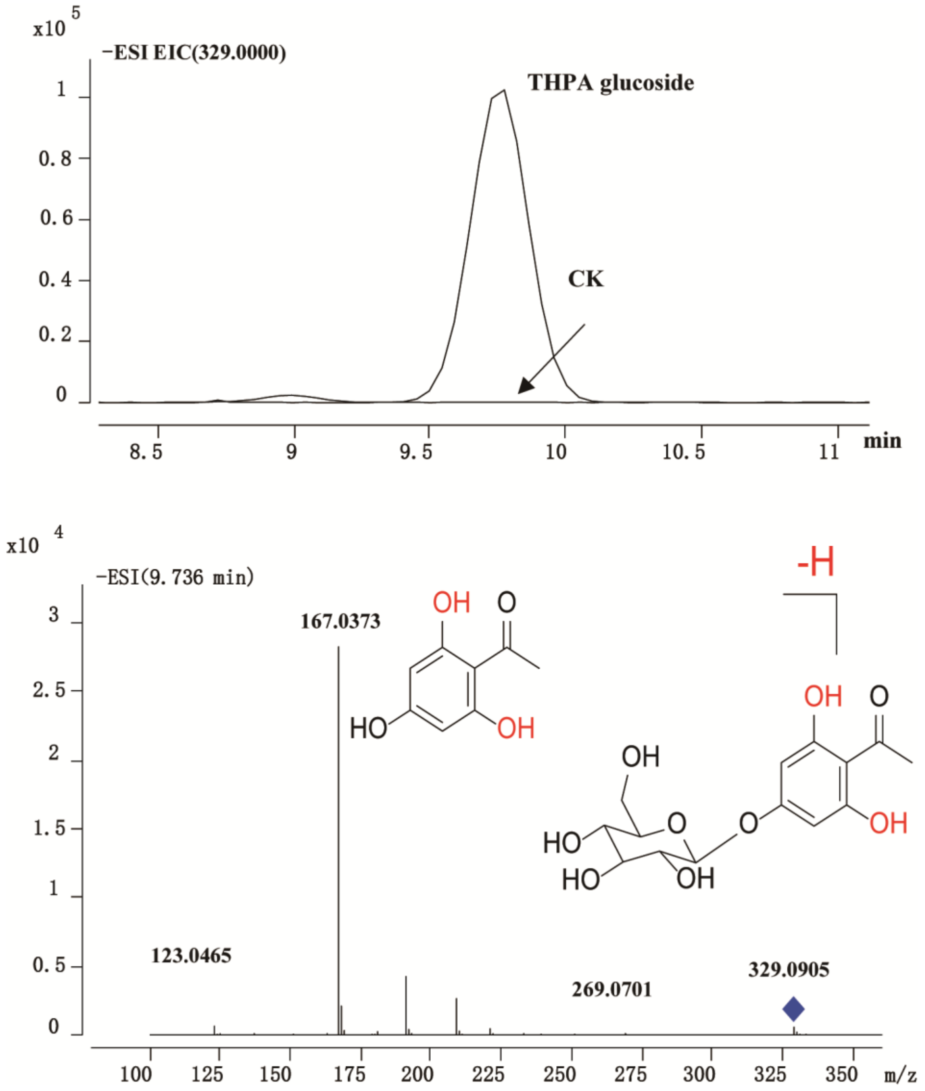

Figure S1. UGT95B17 enzymatic analysis with 2',4',6'-Trihydroxyacetophenone Monohydrate (THPA) and identification of enzymatically formed products by LC–MS.

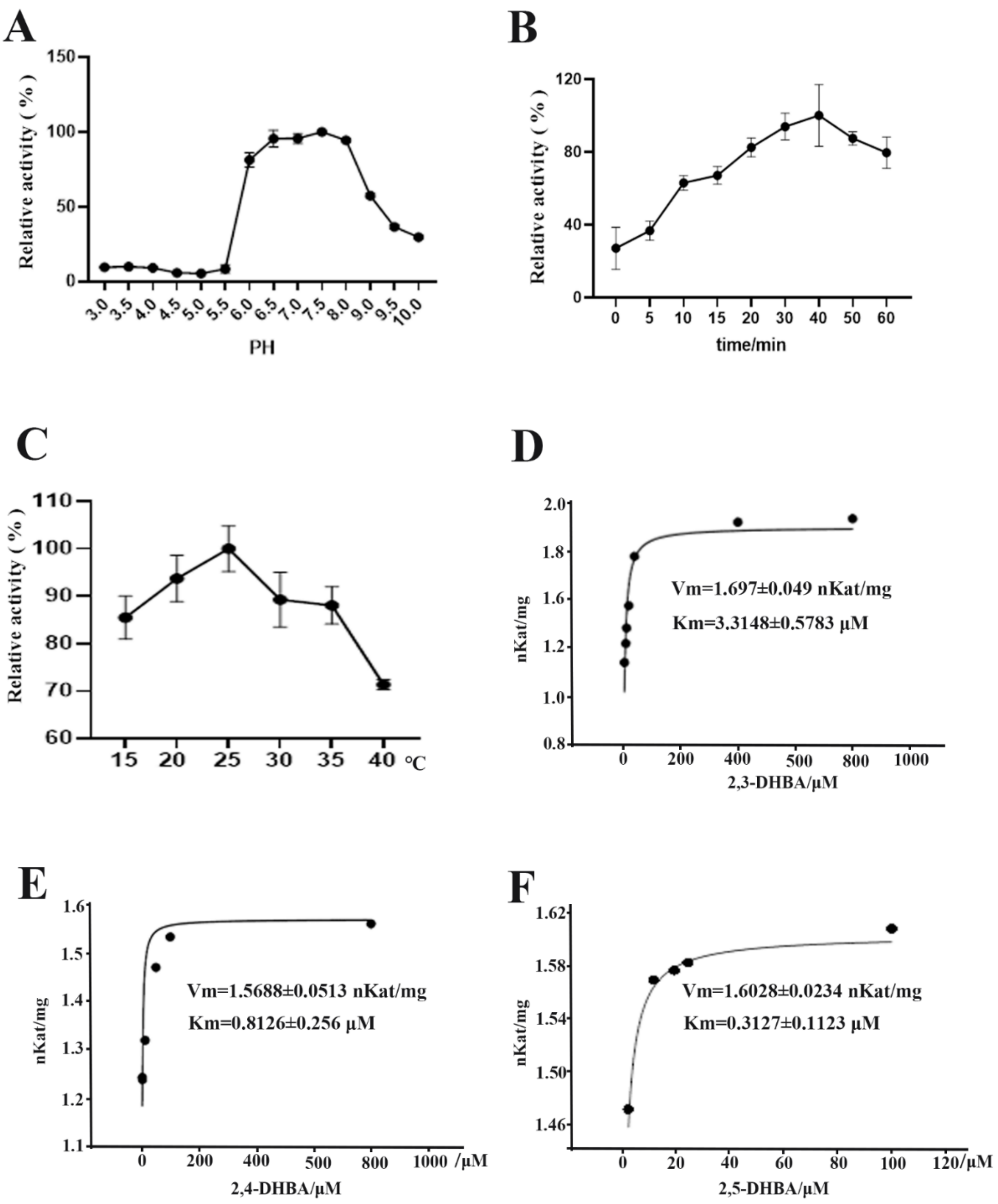

Figure S2. The reaction condition optimization and kinetics of UGT95B17.

A, The pH optimization of UGT95B17. B, The effect of different incubation times on the product formation of UGT95B17 using SA and UDP-glucose as substrates. C, The temperature optimization of UGT95B17. The kinetics of UGT95B17 with 2,3-DHBA (D), 2,4-DHBA (E), 2,5-DHBA (F) was measured under optimized conditions of reaction time.

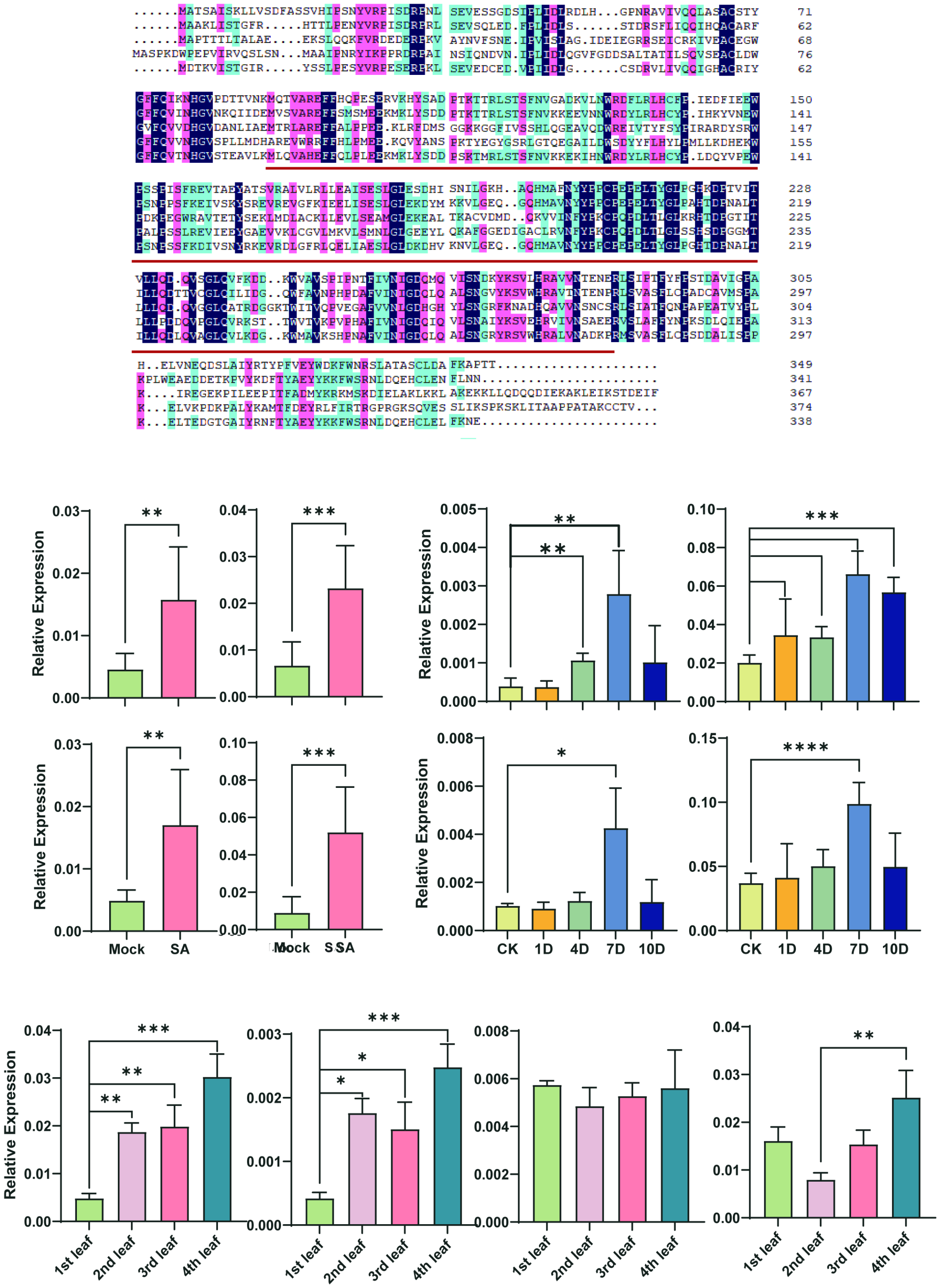

Figure S3. A, Amino acid alignment of deduced CsSH1, CsSH2, S5H (At5g24530), S3H (At4g10500) protein and flavanone 3-hydroxylase enzyme (CsF3H, TEA023790). The red line represents 2OG-Fe(II) oxygenase superfamily (Pfam PF03171 domain). The alignment was done with DNAMAN software (version 7). B, *CsSH1* and *CsSH2* expression were induced by SA. C, Display of *CsSH1* and *CsSH2* expression after inoculation at 1, 4, 7, 10 d. CK stands for uninfected leaves, healthy leaves. D, Gene expression of *CsSH1* and *CsSH2* at four different stages of development (1st, First; 2nd, second; 3rd, third; 4th, fourth).

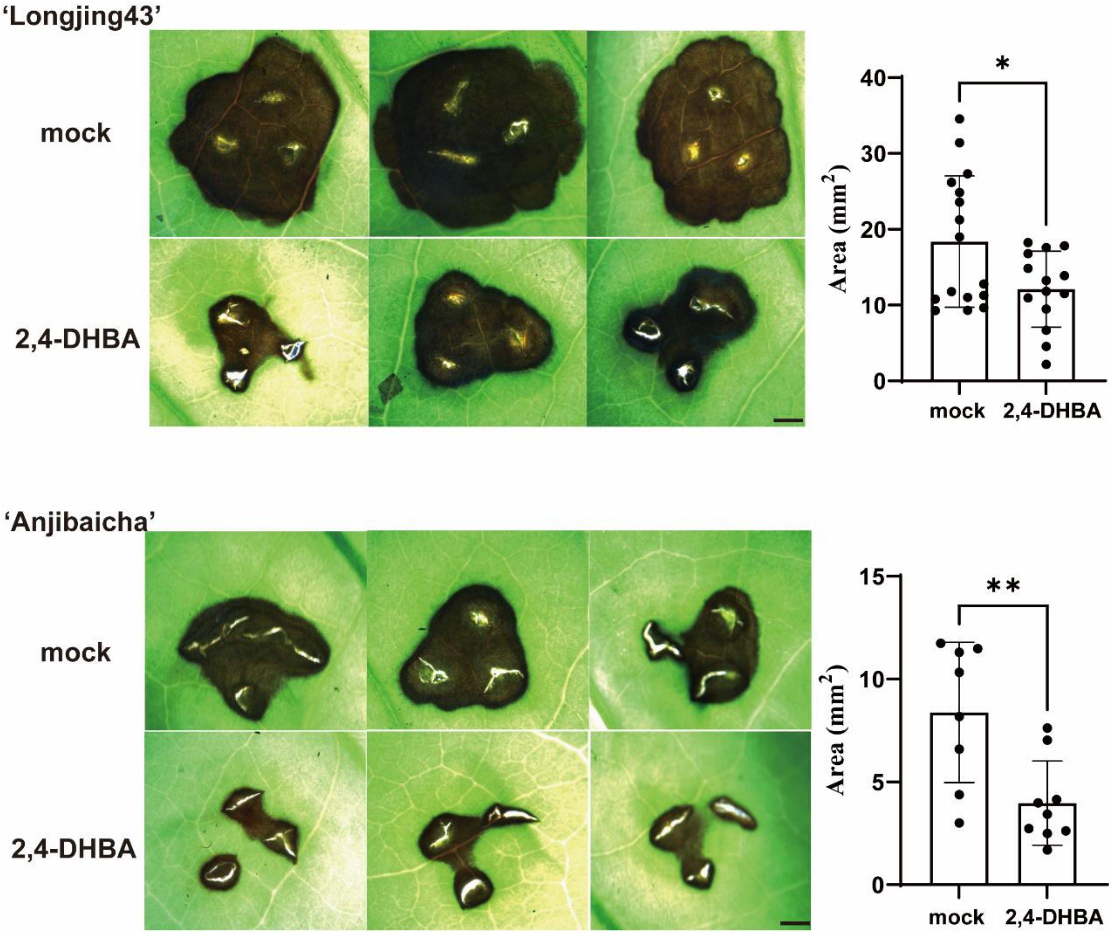

Figure S4. The pathogen symptoms after exogenous application of 2,4-DHBA or water (mock) in tea cultivars ‘Longjing 43’and ‘Anjibaicha’. Bar = 0.1 mm.

Supplemental Table S1 Expression of up-regulated UGTs associated with pathogen-responsive expression in C*amellia sinensis*

id	LB0h1 _fpkm	LB0h2 _fpkm	LB0h3 _fpkm	LB24h1 _fpkm	LB24h2 _fpkm	LB24h3 _fpkm	LB4d1 _fpkm	LB4d2 _fpkm	LB4d3 _fpkm	LB10d1 _fpkm	LB10d2 _fpkm	LB10d3 _fpkm
TEA025984.1	171.45	166.18	168.45	290.51	290.12	289.25	133.91	147.94	139.5	366.1	367.29	320.59
TEA025983.1	67.8	72.86	72.97	99.23	95.55	90.1	84.33	87.05	76.96	183.1	188.91	177.31
TEA012722.1	58.93	51.01	56.29	118.95	123.16	126.69	79.54	79.84	75.53	88.09	75.91	77.18
TEA024806.1	8.15	8.47	6.59	46.03	47.04	45.72	36.32	41.53	42.79	68.51	65.99	58.42
TEA029948.1	18.28	18.38	19.4	28.98	27.47	29.48	146.62	153.97	153.2	63.71	57.1	57.45
TEA006702.1	21.82	21.31	22.33	20.82	19.9	18.8	9.13	10.38	9.43	49.39	49.85	47.48
TEA010338.1	16.79	16.59	17.59	24.26	23.68	20.78	43.47	42.8	45.3	45.86	44.43	41.71
TEA016685.1	13.66	14.22	10.03	14.35	10.84	14.9	7.29	8.47	7.04	34.42	29.35	37.91
TEA001239.1	4.38	2.9	3.43	8.78	8.68	7.95	20.3	19.18	20.32	33.62	38.96	32.19
TEA031489.1	17.13	17.92	19.11	25.15	23.23	25.68	28.09	25.37	28.94	33.27	30.25	31.86
TEA012544.1	5.05	4.5	5.93	4.97	3.09	4.15	2.58	3.24	2.65	33.01	31.52	29.59
TEA006699.1	5.49	7.36	7	15.64	17.81	13.44	10.03	10.08	9.3	25.86	28.04	29.57
TEA025799.1	14.13	12.18	11.67	14.54	14	13.25	11.77	13.13	11.41	27.68	28.35	27.03
TEA022710.1	3.49	3.71	3.85	9.28	10.91	8.24	1.59	1.82	1.91	28.83	29.51	23.73
TEA007509.1	11.98	10.54	8.78	11.7	10.84	11.57	13.88	16.16	15.57	21.2	21.93	23.45
TEA020225.1	9.54	10.9	7.64	12.52	11.65	11.73	9.39	12.64	10.48	22.09	28.57	22.57
TEA006701.1	3.1	0.91	2.58	1.64	3.43	2.04	1.55	1.87	1.11	17.71	25.23	22.09
TEA022516.1	5.25	4.1	4.26	4.94	4.93	4.03	4.35	3.78	2.92	23.43	21.25	20.54
TEA012597.1	2.97	2.29	3.66	12.49	12.16	12.73	2.99	2.68	2.23	21.58	25.75	19.53
TEA000116.1	0.79	0.35	1.06	9.23	9.93	9.99	20.41	19.92	17.85	19.15	16.34	18.64
TEA028429.1	0	0	0	3.22	2.31	1.91	8.5	8.14	10.5	13.54	15.59	18.39
TEA000239.1	11.35	9.01	10.9	22.18	19.91	19.99	17.07	19.02	16.33	18.36	18.01	16.39

TEA030714.1	8.57	7.92	9.88	14.37	16.29	15.39	22.14	21.34	19.83	22.22	16.85	16.11
TEA031670.1	0	0.1	0.32	2.75	2.63	2.54	9.12	8.97	8.59	12.53	17.06	12.97
TEA016355.1	5.14	5.41	5.38	5.81	6.59	6.41	5.56	3.96	3.49	12.02	11.81	12.36
TEA015076.1	3.57	3.76	4.97	11.34	11.01	10.64	15.34	14.11	12.54	12.55	11.25	11.18
TEA024001.1	1.31	0.88	1.54	7.87	8.86	8.54	11.45	12.32	11.73	10.13	9.18	11.17
TEA004424.1	0.75	0.65	0.1	3.26	2.84	3.05	4.45	3.9	4.6	12.22	12.31	9.59
TEA018387.1	0.18	0.21	0.7	3.05	2.74	4.64	11.47	9.06	10.06	9.62	7.8	9.49
TEA001623.1	0	0.15	0.48	0.54	0	0.38	1.33	1.75	2.1	13.4	11.07	9.14
TEA033870.1	0.56	0.9	0.67	2.99	3.5	3.18	3.44	3.58	3.42	9.41	7.54	8.45
TEA029070.1	3.33	4.14	4.36	6.21	6.56	7.52	2.99	2.29	2.69	8.39	7.75	8.06
TEA008897.1	0.25	0	0.95	3.39	4.75	5.58	12.09	9.77	9.78	10.02	6.63	7.66
TEA005008.1	1.49	0.78	0.71	4.44	3.47	3.72	2.9	3.29	2.46	8.7	9.34	7.59
TEA010182.1	1.72	1.59	1.29	1.2	0.84	0.45	0.61	0.13	0.43	9.75	9.31	7.42
TEA006710.1	0.26	2.15	1.56	2.36	2.23	2.09	1.26	1	2.2	7.91	8.23	7.16
TEA006713.1	1.06	1.2	1.91	0.72	0.52	1.02	0.68	0.41	2.13	5.3	5.89	6.57
TEA023526.1	2.92	4.48	3.42	6.65	6.5	5.75	8.09	6.62	7.72	8.29	10.68	6.18
TEA012985.1	20.57	19.8	22.13	15.95	16.49	16.92	52.87	53.7	48.37	5.08	5.03	6.12
TEA033096.1	1.42	2.45	1.7	6.09	4.54	4.84	7.31	8.08	6.87	4.75	6.54	5.94
TEA006704.1	0.42	0.64	0.79	0.68	0.95	0.91	0.2	0.12	0.3	5.9	3.56	5.86
TEA029844.1	1.45	0.86	1.43	3.24	2.8	3.33	2.16	2.63	3.01	7.94	7.41	5.47
TEA004034.1	1.17	1.64	1.31	1.09	0.9	0.84	1.28	0.73	1.37	3.74	4.11	5.33
TEA003466.1	0.76	0.1	0.31	3.74	2.65	3.8	2.08	2.29	2.35	6.07	7.2	4.95
TEA008557.1	0.76	0.68	0.72	2.1	1.21	2.07	6.83	6.11	5.55	4.03	4.95	4.92
TEA002086.1	2.33	3.5	2.81	1.67	1.42	0.93	10.07	10.48	9.38	3.83	1.83	3.77
TEA025120.1	0	0.26	0.42	1.34	1.1	1.68	21.73	25.97	21.97	2.61	2.36	3.39
TEA025792.1	0	0	0	0	0.72	0.36	0	0	0	1.58	2.5	3.32

TEA027948.1	1.78	2.54	3.38	3.34	3.35	4.69	3.64	4.42	3.87	3.44	3.35	3.12
TEA005349.1	0.76	0.47	1.21	0.91	0.37	0.24	0.13	0.19	0.31	2.42	1.78	3.11
TEA007056.1	2.84	1.84	2.47	3.67	3.19	2.79	4.93	4.7	4.38	2.39	3.41	3.09
TEA013923.1	2.95	2.18	2.32	4.34	3	5.1	7.67	10.17	8.83	4.05	3.42	2.83
TEA010914.1	0.26	0.48	0	0.23	1	0.19	2.23	1.38	1.67	3.2	2.76	2.69
TEA025793.1	0	0	0	0	0	0.18	0	0	0	1.56	1.12	2.46
TEA022950.1	0	0.26	0.44	0.74	0.46	0.88	2.09	1.93	2.82	2.01	1.74	2.2
TEA015672.1	0.83	1.2	2.27	3.35	3	2.8	2.65	1.21	2.47	4.14	4.48	2.04
TEA004980.1	1.09	0.47	0.34	1.4	0.88	2.13	5.01	3.93	3.43	2.9	2.3	1.93
TEA006609.1	11.14	11.42	11.18	2.97	2.98	2.64	75.1	80.16	84.75	2.2	1.32	1.71
TEA019162.1	0.08	0	0.1	0.63	0.92	1.04	5.11	5.27	6.27	0.54	1.64	1.71
TEA004486.1	0.26	0.19	0.41	1.35	1	1.74	1.12	0.93	1.1	1.01	1.67	1.65
TEA018897.1	0.57	0.09	0.39	2.7	2.84	2.94	2.31	3.89	2.81	0.96	0.98	1.46
TEA000065.1	0.99	1.04	0.86	0.48	0.47	0.55	1.21	1.24	1.42	0.83	1.2	1.42
TEA019328.1	1.02	0.48	1.53	0.75	0.43	0.25	2.55	2.56	3	1.46	1.49	1.35
TEA008225.1	0.35	0.86	0	0	0.25	0	0	0	0	2.45	1.66	1.24
TEA031409.1	0.07	0	0	1	1.02	0.76	0.43	0.11	0.63	1.12	0.58	1.02
TEA016966.1	0.93	2.02	1.74	1.89	1.48	1.88	2.31	2.14	2.35	1.61	1.23	0.98
TEA027943.1	0.21	0.24	0.27	0.28	1.21	1.29	1.07	0	0.53	0.24	0.77	0.94
TEA007328.1	0	0.23	0.26	0.5	0.13	0.12	0.95	0	0.41	0.67	0.9	0.82
TEA019941.1	0	0	0	0.04	0	0	0.41	0.51	0.45	0.71	1.04	0.74
TEA030867.1	0	0	0.31	0.12	0.06	0.12	0.26	0	0.13	0.73	0.86	0.67
TEA013757.1	0	0	0	0.13	0.14	0.24	0.16	0	0.08	0.11	0.44	0.64
TEA002066.1	0.17	0.19	0.21	0.12	0.44	0.19	2.31	2.93	2.72	0.46	0.8	0.58
TEA026194.1	0	0.23	0	0.42	0.21	0.76	0.81	1.41	1.1	0	0.65	0.43
TEA022238.1	0	0	0.1	0.18	0	0.06	0.93	0.67	0.71	0.37	0.24	0.39

TEA000117.1	0.07	0.08	0.09	0.55	0.54	0.53	0.93	0.91	1.08	0.32	1.22	0.38
TEA033357.1	0	0	0	0.14	0	0	0	0.79	0.54	0.55	0.09	0.35
TEA031756.1	0	0	0	0.13	0.38	0.21	0.6	0.68	0.37	0.21	0.42	0.33
TEA031181.1	0	0	0.51	0.58	0.57	0.62	0.66	0.35	1.77	0.23	0.28	0.24
TEA000079.1	0.55	0	0.34	0.09	0.54	0.22	0.09	1.84	0.94	0.73	0.15	0.22
TEA002089.1	0	0	0	0.12	0.06	0.06	2.73	2.75	3.28	0	0	0.2

Supplemental Table S2 Pearson correlation analysis

		2,3-DHBA Glycoside	X-DHBA Glycoside	2,5-DHBA Glycoside
TEA025984.1	correlation coefficient	0.523	0.33	0.749**
	p value	0.081	0.295	0.005
TEA025983.1	correlation coefficient	0.644*	0.650*	0.702*
	p value	0.024	0.022	0.011
TEA012722.1	correlation coefficient	0.481	-0.069	0.567
	p value	0.113	0.83	0.054
TEA024806.1	correlation coefficient	0.916**	0.633*	0.839**
	p value	0	0.027	0.001
TEA029948.1	correlation coefficient	0.477	0.464	0.021
	p value	0.117	0.129	0.949
TEA006702.1	correlation coefficient	0.318	0.436	0.516
	p value	0.314	0.157	0.086
TEA010338.1	correlation coefficient	0.785**	0.768**	0.436
	p value	0.002	0.004	0.156
TEA016685.1	correlation coefficient	0.387	0.424	0.593*
	p value	0.215	0.17	0.042
TEA001239.1	correlation coefficient	0.785**	0.846**	0.602*
	p value	0.002	0.001	0.038
TEA031489.1	correlation coefficient	0.868**	0.692*	0.683*
	p value	0	0.013	0.014

* $p<0.05$ ** $p<0.01$

Supplemental Table S3 Lists of Primers used in the study

	Sequenees(5'-3')
TEA025983-F	gatctggttccgcgtggatccATGGCAGTAGATGGAGAGATCTTAATC
TEA025983-R	ggccgctcgagtcgacccgggTCAAGGTGATTTTTGGCTAATAAAA
TEA025983-AsODN	GCTCTATGCAGGGGAAGAGG
TEA025983-RTF	CGAAATCACCGACGATGC
TEA025983-RTR	TTGCTCCAGCTCATCATCAC
TEA025983-RTF-1	CGAAATCACCGACGATGCC
TEA025983-RTR-1	GGGTCGGGTGGGATTGTTAA
TEA025983-mCherry-F	gcttctgtatattctgcccaaattcgcgACTAGTATGGCAGTAGATGGAGAGAT
TEA025983-mCherry-R	ctcctcgcccttgctcaccatACTAGTAGGTGATTTTTGGCTAATAAAATCCCT
qPR1-F	GACAGTGTCGGATGCAGTGA
qPR1-R	TTCTGGCACTGAACCCTAGC
qPR2-F	CTCTGTTCACAGCTCCCTCG
qPR2-R	GTTCCACCAACTGTAGGCCA
qPR5-F	GGCTGCAACTTCGATTCCT
qPR5-F	AGGGTTTCACTCCCATACCC
qICS1-F	GAACGGCTAGCCAGTGAAGT
qICS1-R	AGTTGCTTCCACAGAGAGCC
qEDS1-F	CGACATCGTCCCTCGCATAA
qEDS1-R	CGGATTTGTGGACCCCATCA
qPAD4-F	ATGTTTCTGGGCGAGAGTGG
qPAD4-R	CTCCCCATTCCTTCACCCAC
GAPDH-F	TTGGCATCGTTGAGGGTCT
GAPDH-R	CAGTGGGAACACGGAAAGC
TEA002405 -RTF	GGCGATAAACTCGATCCAAA
TEA002405 -RTR	TGGCGTAAACCTGCTTCTCT
TEA002405 –RTF-1	AGCAGAGGTGGTGAAGCTGT

TEA002405 –RTR-1	GAAGACAGCCCCAGAGTGAG
TEA002405-AsODN-1	CGTCGAAAGTCATGGCCTTG
TEA002405-AsODN-2	TCGTCGAAAGTCATGGCCTT
TEA027220-RTF	GGCCTTGACAAAGACCATGT
TEA027220-RTR	GGATTTGACAGCCATCCACT
TEA027220-RTF-1	TGGCACATGAATTTTTCCAA
TEA027220-F	gatctggttccgcgtggatccATGGATACGAAGGTCATCTCCACC
TEA027220-R	ggccgctcgagtcgacccgggTTATTCGTTCTTGAATAGTTCCAAACA
TEA002405 -F	gatctggttccgcgtggatccATGGCTAGCCCAAAAGATTGG
TEA002405 –R	ggccgctcgagtcgacccgggTCAGACAGTACAACACTTAGCAGTAGCA
TEA025983-1302F	acgggggactcttgaccatggATGGCAGTAGACGGAGAGATC
TEA025983-1302R	aagttcttctcctttactagtTCAAGGTGATTTTTGGCTAATAAAATCCC

Supplemental Table S4 List of selected plant glycosyltransferases in the phylogenetic tree.

gene name	Match in NCBI and TPIA
UGT74F1	At2g43840
UGT75B1	At1g05560
UGT89A2	At5g03490
UGT76D1	At2g26480
UGT71C3	At1g07260
UGT71C5	OAP14418.1
UGT84A13	AHA54051.1
UGT84B1	At2g23260
UGT74E2	At1g05680
UGT78K1	ADC96620.1
UGT73C6	OAP07438.1
UGT79B2	AT4G27560
UGT79B3	AT4G27570
*Cs*UGT75L12	ALO19892.1
*Cs*UGT91Q2	TEA006435
*Cs*UGT78A14	ALO19888.1
*Cs*UGT78A15	ALO019889.1
*Cs*UGT87E7	TEA006702
UGT95A1	ACB56927.1
UGT95B7	QDM38906.1
UGT7	BBC62108.1

Supplemental Table S5 Enzyme activity assay toward DHBA of TEA008557, TEA029948, TEA031670.

	TEA008557					TEA029948					TEA031670				
Substrate	xyl	gal	gla	rha	glc	xyl	gal	gla	rha	glc	xyl	gal	gla	rha	glc
2,3-DHBA	×	×	×	×	×	×	×	×	×	×	×	×	×	×	×
2,4-DHBA	×	×	×	×	×	×	×	×	×	×	×	×	×	×	×
2,5-DHBA	×	×	×	×	×	×	×	×	×	×	×	×	×	×	×
2.6-DHBA	×	×	×	×	×	×	×	×	×	×	×	×	×	×	×
